# Optimizing data integration improves gene regulatory network inference in *Arabidopsis thaliana*

**DOI:** 10.1093/bioinformatics/btae415

**Published:** 2024-06-24

**Authors:** Océane Cassan, Charles-Henri Lecellier, Antoine Martin, Laurent Bréhélin, Sophie Lèbre

**Affiliations:** LIRMM, Univ Montpellier, CNRS, Montpellier, 34095, France; LIRMM, Univ Montpellier, CNRS, Montpellier, 34095, France; IGMM, Univ Montpellier, CNRS, Montpellier, 34090, France; IPSIM, CNRS, INRAE, Institut Agro, Univ Montpellier, 34060, Montpellier, France; LIRMM, Univ Montpellier, CNRS, Montpellier, 34095, France; LIRMM, Univ Montpellier, CNRS, Montpellier, 34095, France; IMAG, Univ Montpellier, CNRS, Montpellier, 34090, France; Université Paul-Valéry-Montpellier 3, Montpellier, 34090, France

## Abstract

**Motivations:**

Gene regulatory networks (GRNs) are traditionally inferred from gene expression profiles monitoring a specific condition or treatment. In the last decade, integrative strategies have successfully emerged to guide GRN inference from gene expression with complementary prior data. However, datasets used as prior information and validation gold standards are often related and limited to a subset of genes. This lack of complete and independent evaluation calls for new criteria to robustly estimate the optimal intensity of prior data integration in the inference process.

**Results:**

We address this issue for two regression-based GRN inference models, a weighted random forest (weigthedRF) and a generalized linear model estimated under a weighted LASSO penalty with stability selection (weightedLASSO). These approaches are applied to data from the root response to nitrate induction in *Arabidopsis thaliana.* For each gene, we measure how the integration of transcription factor binding motifs influences model prediction. We propose a new approach, DIOgene, that uses model prediction error and a simulated null hypothesis in order to optimize data integration strength in a hypothesis-driven, gene-specific manner. This integration scheme reveals a strong diversity of optimal integration intensities between genes, and offers good performance in minimizing prediction error as well as retrieving experimental interactions. Experimental results show that DIOgene compares favorably against state-of-the-art approaches and allows to recover master regulators of nitrate induction.

**Availability and implementation:**

The R code and notebooks demonstrating the use of the proposed approaches are available in the repository https://github.com/OceaneCsn/integrative_GRN_N_induction

## 1 Introduction

Gene regulatory networks (GRNs) inference has the objective of describing the relationships between genes in the context of transcription, which can provide invaluable insight into environmental adaptation or developmental processes in living organisms. To reconstruct these networks, statistical GRN inference methods usually leverage high-throughput genomics. A well-established input is transcriptomic data, as it provides genome-wide measures of gene expression and has become increasingly available. Regression-based techniques for GRN inference are a popular class of methods, that have shown great performances in benchmarks like DREAM (Marbach *et al.* 2012a). They rely on the assumption that the expression of regulator genes can be used to predict the expression of their target genes in a set of relevant experimental conditions. Once regression models are fit, they allow the extraction of the influence of each regulator over each target gene, and the strongest regulator–target gene pairs are assembled to form a final sparse GRN. Regression-based inference algorithms mainly differ in their choice of regression function to link the expression of a target gene to the expression of its regulators. For example, TIGRESS (Haury *et al.* 2012), MEN (Greenfield *et al.* 2013), or The INFERELATOR (Skok-Gibbs *et al.* 2022) techniques implement linear and often regularized models for this task, while GENIE3 (Huynh-Thu *et al.* 2010) and inspired works (Petralia *et al.* 2015, Huynh-Thu and Geurts 2018, Cirrone *et al.* 2020, Cassan *et al.* 2021) model non-linear relations *via* random forests (RFs) or, more broadly, ensembles of trees.

Given the under-determined nature of GRN inference from expression alone, using additional sources of data can guide the choice between several regulators explaining expression data equally well. Complementary omics have already been used in addition to gene expression to enhance GRN inference, such as TF binding experiments (mostly ChIP-Seq) or transcription factor binding motifs (TFBM) (Kundaje *et al.* 2007, Marbach *et al.* 2012b, Qin *et al.* 2014, Aibar *et al.* 2017, Cirrone *et al.* 2020, Clercq *et al.* 2021, Skok-Gibbs *et al.* 2022), knock-outs and protein–protein interactions (Petralia *et al.* 2015), or chromatin accessibility (Miraldi *et al.* 2019, Clercq *et al.* 2021).

In a linear context, prior information can be integrated during the estimation of regularized models by modulating the penalty strength for each TF with a weighted version of the LASSO (Tibshirani 1996, Bergersen *et al.* 2011) or its variations [e.g. the ElasticNet (Zou and Hastie 2005, Christley *et al.* 2009, Greenfield *et al.* 2013, Miraldi *et al.* 2019, Skok-Gibbs *et al.* 2022], or by making use of a Bayesian prior (Greenfield *et al.* 2013, Siahpirani and Roy 2017, Skok-Gibbs *et al.* 2022). In this framework, the strength chosen for the penalty is an important feature of the method. An in-depth study introducing the method MEN (Greenfield *et al.* 2013), a weighted ElasticNet, explored a resolutive range of data integration strengths and then choose an integration strength maximizing effective data integration. More recently, the mLASSO-StARS approach (Miraldi *et al.* 2019) was introduced to select a small subset of robust regulators for each target gene in oriented GRN inference by adapting the StARS approach (Liu *et al.* 2010) for the LASSO, while integrating prior complementary data. In that work, the integrated priors were TFBMs in accessible chromatin. Three values of prior reinforcement were investigated, and the one that maximized the area under the precision and recall curve against a CHIP-Seq gold standard was selected.

Regarding non-linear regression, iRafNet (Petralia *et al.* 2015) proposed a RF based procedure. It consists in weighting the random sub-sampling of regulators during trees elongation, so that regulators supported by prior knowledge are more likely to get chosen at decision nodes. In the iRafNet method, the weights controlling the contribution of prior data to expression are provided by a predefined function, specific to each type of prior, but without specific tuning. This strategy was further adapted to time series expression data in OutPredict (Cirrone *et al.* 2020), a dynamic extension of both GENIE3 (Huynh-Thu and Geurts 2018) and iRafNet (Petralia *et al.* 2015).

Existing integrative regression models thus have a great potential to predict GRNs from several types of prior complementary omics. However, the extent to which prior complementary data should contribute to inferred GRN models relatively to gene expression data remains difficult to estimate, especially when prior data is noisy, incomplete or when it contradicts gene expression data. Yet, fine tuning the contribution of prior information to gene expression data was rarely explored in past works. When it was, the choice of prior integration strength relied on a gold standard that is either identical to the integrated prior like in MEN (Greenfield *et al.* 2013), or of a related nature [in the mLASSO-StARS paper, the prior information of TFBMs is necessarily correlated to ChIP-seq validation data (Miraldi *et al.* 2019)]. This highlights the need for a more robust and independent calibration of prior data integration strength. In fact, while the integrated prior data is gene-specific in nature, the strength or importance with which it should contribute to GRN inference has not been tuned specifically for each target gene by previous approaches (Greenfield *et al.* 2013, Petralia *et al.* 2015, Siahpirani and Roy 2017, Miraldi *et al.* 2019, Cirrone *et al.* 2020, Skok-Gibbs *et al.* 2022). This is especially important because prior knowledge is not always relevant to the condition at hand, and this relevance can vary from gene to gene. For the same reason, gold standard data like known regulator–gene interactions may also be irrelevant and should be avoided when choosing the importance of prior data.

To address these limitations, we propose a new optimization scheme, DIOgene (data integration optimization for gene networks), which is based on gene-specific measures of effective data integration, gene expression prediction accuracy, and a simulated null hypothesis. The aforementioned fallibility of TF-target gold standards and their proximity with prior data made us prefer, as a tuning and evaluation metric, the accuracy of regression models in their supervised prediction task (the MSE). This metric has the advantage, unlike many gold standards, to be specific to the conditions and cell lines used for transcriptome collection, and can be measured for all genes. More generally, any causal model should be predictive, which is why we closely monitor the prediction performance of regression models for GRN tuning and evaluation (Shen *et al.* 2024).

Moreover, in order to represent the most common methods in the field of integrative GRN inference, we illustrate our results using a weightedLASSO and weightedRF model, that are unified re-implementation of existing algorithms both in the linear and non-linear cases (Bergersen *et al.* 2011, Greenfield *et al.* 2013, Petralia *et al.* 2015, Miraldi *et al.* 2019, Skok-Gibbs *et al.* 2022). We optimize their level of data integration with DIOgene to model the transcriptomic response to nitrate induction in the roots of *Arabidopsis thaliana* (Varala *et al.* 2018) using TFBMs in target gene promoters as prior information. Our results, when compared to existing algorithms, illustrate that a gene-specific modulation of data integration can be more profitable than enforcing data integration in an indiscriminate fashion. With this case study, we hope to open a reflection about data integration and evaluation practices in the field.

## 2 Materials and methods

### 2.1 Expression, prior, and validation datasets

As a case study for GRN inference, we chose the transcriptomic root response to nitrate induction in the model plant *A.thaliana* (Varala *et al.* 2018). This dynamic response was already well characterized, and used in other previous developments to chart regulatory networks (Varala *et al.* 2018, Brooks *et al.* 2019, Cirrone *et al.* 2020). Continuing efforts to uncover these regulatory mechanisms is of great agricultural interest, as nitrate is the main source of nitrogen used by most plants. Gene expression was measured in seedling roots at 0, 5, 20, 30, 45, 60, 90, and 120 min after nitrate or control treatments (*N *=* *45 samples). We selected differentially expressed genes responding to nitrate induction in time by testing the interaction terms between nitrate treatment and time modeled as natural splines (Supplementary Method A, Fig. S1a). These nitrate-responsive genes and regulators are taken as input for GRN inference (Supplementary Tables S1 and S2). TFBMs, encoded by position weight matrices (PWMs), were retrieved from the JASPAR (Castro-Mondragon *et al.* 2021) and Plant Cistrome (O’Malley *et al.* 2016) databases and searched in Arabidopsis promoters to serve as prior information for GRN inference (Supplementary Method A, Fig. S1b and c). Finally, we also leveraged the *in vitro* binding events from DAP-Seq experiments as a partial and condition-agnostic gold standard to evaluate the predicted GRNs edges a posteriori (O’Malley *et al.* 2016). Like any other gold standard, DAP-seq data have certain technical limitations, but compared to alternative approaches (e.g. ChIP-seq), it can be more easily scaled to a larger number of TFs (Bartlett *et al.* 2017).

### 2.2 Integrative GRN models

To address both the linear and non-linear cases, we adapted, from existing algorithms, (Bergersen *et al.* 2011, Greenfield *et al.* 2013, Petralia *et al.* 2015, Miraldi *et al.* 2019, Skok-Gibbs *et al.* 2022) two integrative regression-based GRN inference procedures for this study, namely weightedLASSO and weightedRF. These two approaches use the expression levels of regulator genes to predict the expression of target genes, but with additional modeling that prioritizes the use of regulators with a TFBM in the target gene promoter during model estimation.

The TFBM information is encoded in a prior matrix Π that gives a prior value $\Pi_{r,t}\in [0,1]$ for each regulator–target gene pair (*r*, *t*):

(1) $$\Pi_{r,t}=\{\begin{array}{c} 0:\text{if the motif of}\;r\;\text{is not in the promoter of}\;t\\ 1:\text{if the motif of}\;r\;\text{is in the promoter of}\;t\\ \frac{1}{2}:\text{if the motif of}\;r\;\text{is unknown} \end{array}$$

Throughout this study, a parameter *α* is used to tune data integration strength: its value ∈[0,1] controls the contribution of TFBM information to expression data. At *α* = 0, no TFBM information is used for selecting the regulators, i.e. expression alone is used, while at *α* = 1, only regulators possessing a TFBM in the target gene can be used in the regression model. Briefly, this is done by reducing the penalty strength of TFBM-supported TFs in weightedLASSO and by weighting the random sampling of variables in favor of TFBM-supported TFs in the regression trees elongation of weightedRF. The definition of the weights for integrating TFBM prior information $\Pi_{r,t}$ for a given *α*, as well as the estimation procedures for weightedLASSO and weightedRF are detailed in Supplementary Methods B and C, and illustrated in Supplementary Fig. S2a–c.

### 2.3 Gene-specific optimization of *α* (DIOgene)

Choosing the value of *α* is instrumental: it reflects strong modeling assumptions and has tangible impacts on inferred GRNs. In order to measure the direct consequence of modulating data integration through *α*, we introduce the notion of effective data integration (EDI), that reflects the importance of TFBM-supported regulators in the predictions of a regression model. Here, the importance of a regulator is estimated by the classical “mean decrease accuracy” approach proposed by Breiman (Breiman 2001), which is measured by the model performance loss when the expression of this regulator is shuffled (Supplementary Methods D and E). For a target gene *t*, regulators are ranked by increasing values of importance, and the EDI is the average position in this ranking of TFBM-supported regulators, i.e. the regulators for which $\Pi_{r,t}=1$

(2) $${\text{EDI}}_{t\alpha }=\frac{\Sigma_{\Pi_{r,t}=1}\text{Rank}({\text{Importance}}_{rt\alpha })}{{\#}_{\Pi_{r,t}=1}}$$

EDI is close to 1 (resp. *R*, the total number of regulators) when all regulators with a motif have low (resp. high) importance. We expect that increasing *α* will increase the importance values of TFBM-supported regulators, and thus increase EDI. For the occasional target genes with no TFBMs in their promoter (10 out of the 1426 nitrate-responsive genes), EDI cannot be computed and no data integration is done in DIOgene, i.e. their *α* value is automatically set to 0.

Given that enforcing data integration interferes with model estimation based solely on error minimization, a loss of prediction accuracy can also be expected from increasing EDI. The foundation of DIOgene is that we should integrate prior TFBMs information only when it does not induce a major deterioration of prediction performance, which is measured by the model mean square error (MSE) on unseen samples (Supplementary Method D).

In order to define what is an acceptable loss of MSE, we create a synthetic null hypothesis that provides a reference for comparison. In this simulated null dataset, we break the link between gene expression and TFBM information by randomly unmatching the expression profiles between regulators. A regulator then keeps its correct TFBMs, but is attributed the wrong expression profile. In such a synthetic baseline, there is theoretically no joint information to be learned from the combination of expression and TFBMs, and increasing data integration strength can only provide uninformative TF-target gene interactions.

In order to identify the appropriate amount of TFBMs knowledge to inform GRN inference, we propose that the optimal value of *α* for target gene *t* (hereafter denoted as $\alpha_{t,\text{opt}}$), is chosen where true prediction error is most reduced as compared to the error committed under the simulated null hypothesis (*H*_0_). This corresponds to a level of data integration where TFBM incorporation in the model provides a sufficient improvement of prediction over the error expected under *H*_0_. Formally, the normalized difference in MSE between true and randomized datasets for a value of *α* is measured by the Student statistic

(3) $$T_{t\alpha }=\frac{\mu_{\text{MSE}}(EDI_{t\alpha })-\mu_{{\text{MSE}}_{0}}(EDI_{t\alpha })}{\sqrt{\frac{\sigma_{\text{MSE}}{\left(EDI_{t\alpha }\right)}^{2}+\sigma_{{\text{MSE}}_{0}}{\left(EDI_{t\alpha }\right)}^{2}}{N}}}$$

with $\mu_{\text{MSE}}(EDI_{t\alpha })$ the mean MSE at $EDI_{t\alpha }$, $\sigma_{\text{MSE}}(EDI_{t\alpha })$ the standard deviation of MSE at $EDI_{t\alpha }$, $\mu_{{\text{MSE}}_{0}}(EDI_{t\alpha })$ the mean MSE on the null dataset interpolated at $EDI_{t\alpha }$, and $\sigma_{{\text{MSE}}_{0}}(EDI_{t\alpha })$ the standard deviation of MSE on the null dataset interpolated at $EDI_{t\alpha }$. *N* is the number of repetitions of weightedLASSO or weightedRF performed in order to estimate the MSE and its dispersion for each value of *α*, on the true and shuffled datasets. In weightedLASSO, variability stems from the cross-validation partitionings used to optimize the LASSO regularization parameter, and from the bootstrapping layer we introduced to improve stability (Supplementary Method B). In weightedRF, variation classically stems from the random sampling of TFs at each node of the trees, and from the bootstrapped sample used to grow each tree. Additionally, the variation observed on the shuffled dataset is due to the different permutations representing *H*_0_. For each value of *α*, the Student statistics $T_{t\alpha }$ are then compared to a Student distribution of 2N−2 degrees of freedom, and provide a list of (FDR-adjusted) *P*-values $p_{t\alpha }$. $\alpha_{t,\text{opt}}$ is then the value of *α* that minimizes $p_{t\alpha }$ on the condition that at least one value of *α* provides an adjusted *P*-value lower than 5%:

(4) $$\alpha_{t,\text{opt}}=\{\begin{array}{cc} 0 & \text{if}\;\underset{\alpha \in [0,1]}{\text{min}}(p_{t\alpha })>0.05\\ \underset{\alpha \in [0,1]}{\text{argmin}}\;(p_{t\alpha }) & \text{otherwise}. \end{array}$$

When the minimum *P*-value is greater than 5%, we consider that no level of data integration is appropriate and $\alpha_{t,\text{opt}}$ is set to 0.

### 2.4 GRN construction and evaluation

Once the values of $\alpha_{t,\text{opt}}$ have been selected, a regression model is learned for each target gene. All these models constitute a complete GRN, with many regulators per target gene. To measure the quality of this complete GRN, we relied on the median MSE computed on all target genes (Supplementary Method D). Studies of known GRNs usually report low values of edge density, typically between 0.001 and 0.1 (Leclerc 2008, Hayes *et al.* 2013, Campos and Freyre-González 2019, Koutrouli *et al.* 2020). A classical strategy in GRN inference is therefore to select the most important edges satisfying a biologically relevant user-specified network density. This is done in DIOgene by selecting only the most important pairs of regulator–target genes based on the importance metric (the mean decrease accuracy, Supplementary Method E). Density is defined as $D=\frac{E}{E_{\mathrm{total}}}$, with *E* being the number of edges in the inferred network, and $E_{\mathrm{total}}=R(T-1)$ being the total number of edges in a complete oriented GRN containing *R* regulators and *T* genes (Cassan *et al.* 2021). The number of top-ranked edges to select in order to satisfy a density *D* is thus

(5) E=⌊DR(T−1)⌋.

Once a sparse GRN of the chosen density has been built, it can be compared to a gold standard network (in our case DAP-Seq data) based on precision and recall metrics. Let us consider G as a set of experimentally observed regulatory interactions (gold standard) restricted to interactions involving genes given as input for GRN inference. E is the set of inferred interactions restricted to TFs studied in the gold standard. The other inferred interactions can neither be confirmed nor falsified and are thus not taken into account here. Precision (the fraction of edges in E present in G) is defined as $\frac{\left|E\cap G\right|}{\left|E\right|}$ while recall (the fraction of edges in G retrieved by E) is defined as $\frac{\left|E\cap G\right|}{\left|G\right|}$.

## 3 Results

### 3.1 Optimal TFBMs integration strength differs strongly between target genes

**Figure 1.**
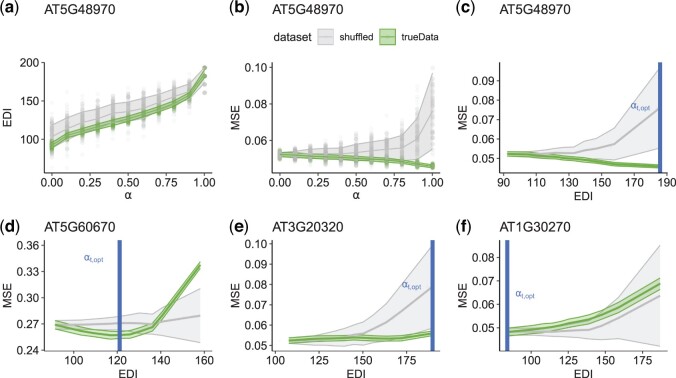
Gene-specific data integration with DIOgene is tuned by monitoring model performance variation relatively to a synthetic null hypothesis. For the target gene AT5G48970: (a) the EDI depending on *α*, (b) the MSE depending on *α*, (c) the MSE depending on EDI (from panels a and b). The proposed gene-specific $\alpha_{t,\text{opt}}$ (vertical line) is the value for which the MSE is most reduced as compared to the randomized baseline [Equations (3) and (4)]. (d–f) The MSE depending on EDI for three other gene examples, representing different scenarios of data integration and thus different values of $\alpha_{t,\text{opt}}$. The trends are shown for weightedRF on true data and shuffled datasets where TF expression profiles were randomly unmatched from their motif. For each value of *α*, 100 models were run and the standard deviation around the mean is represented. The MSE is normalized by the variance of the target gene expression. Similar scenarios emerge in the linear model weightedLASSO (Supplementary Fig. S3).

We ran weightedRF (100 repetitions) and weightedLASSO (50 repetitions) for global values of *α*, ranging from 0 to 1 with a step of 0.1. First, we confirm that both weightedRF and weightedLASSO effectively incorporate TFBM information during their estimation, attributing higher importance measures to TFBM-supported variables as *α* increases. This is supported by EDI curves smoothly increasing with *α* [see the gene examples in Fig. 1a and Supplementary Fig. S3 (left column), and the global picture in Supplementary Fig. S4a]. When applying a density threshold to build sparse GRNs, we also observe that increasing *α* leads to the selection of edges with more and more TFBM support. At *α *= 1, which is the maximal level of data integration, TFBM support equals 1. This means that inferred GRNs are restricted to interactions supported by a TFBM (Supplementary Fig. S4b).

**Figure 2.**
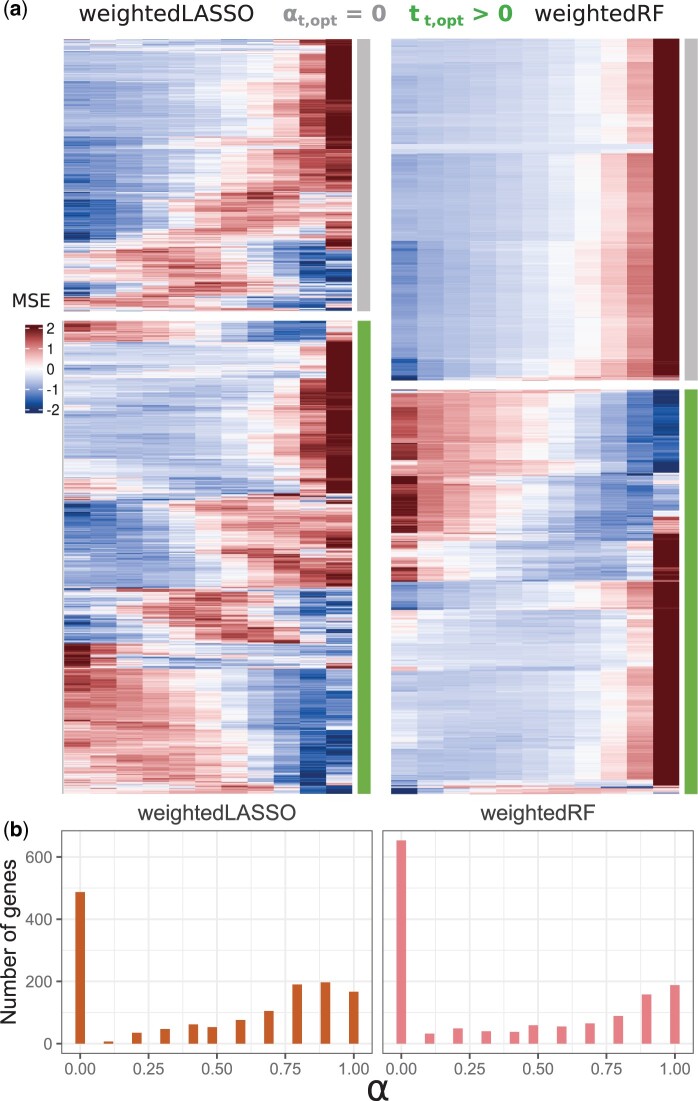
Gene-specific tuning of TFBM integration in the 1426 nitrate-responsive target genes with DIOgene leads to diverse MSE behaviors and integration intensities. (a) Scaled MSE (*z*-score) in weightedLASSO and weightedRF on true data depending on *α* for two types of genes: target genes with $\alpha_{t,\text{opt}}=0$ and target genes with $\alpha_{t,\text{opt}}>0$. (b) Distribution of $\alpha_{t,\text{opt}}$ values for the 1426 nitrate-responsive target genes in weightedLASSO and weightedRF.

An overview of the MSE profiles depending on *α* for all nitrate-responsive genes reveals a lot of diversity in how model performance can be driven by data integration strength, foreshadowing the usefulness of a gene-level procedure [see the gene example in Fig. 1b and Supplementary Fig. S3 (middle column), and the global picture in Fig. 2a]. We thus applied DIOgene to optimize TFBM integration in weightedLASSO and weightedRF at the target gene level [Equations (3) and (4)]. This confirmed that depending on the target genes, enforcing data integration has different effects on the predictive capabilities of the regression models, both in absolute error and relatively to the simulated null hypothesis (see four examples in Fig. 1c–f). Very interestingly, for several genes like AT5G48970, enforcing data integration leads to a reduced MSE on test samples (Fig. 1c). This illustrates that data integration can effectively guide the choice of variables toward more robust and meaningful regulators, allowing the model to better predict target gene expression in unseen conditions. In this case, data integration can often be pushed to its maximal intensity, given that the maximal divergence from the simulated null data occur at $\alpha_{t,\text{opt}}=1$. For several other target genes, e.g. AT5G60670 (Fig. 1d), the strongest improvement over the randomized baseline is achieved for an intermediate value of *α* (0.5 in Fig. 1d). For genes like AT3G20320, there is no reduction of MSE induced by data integration, however DIOgene sets $\alpha_{t,\text{opt}}$ to 1 because the MSE increase remains low in comparison to the randomized baseline (Fig. 1e). Finally, the MSE of target genes can be increased by TFBM incorporation in the same proportion as in the simulated null data, like for instance AT1G30270, where $\alpha_{t,\text{opt}}$ is set to 0 by our procedure (Fig. 1f).

The application of DIOgene to all nitrate-responsive target genes led to one $\alpha_{t,\text{opt}}$ value per target gene. Among the 1426 input target genes, the number of genes for which TFBM information is integrated to expression, i.e. $\alpha_{t,\text{opt}}>0$, was 939 for weightedLASSO and 773 for weightedRF. The distribution of $\alpha_{t,\text{opt}}$ for the 1426 nitrate-responsive genes reveals that, similarly for the two models, there is also a large pool of genes that do not benefit from data integration according to our criterion (487 and 653 for weightedLASSO and weightedRF, respectively, Fig. 2b). This suggests that data integration can often lead to a significant deterioration of model predictive capabilities as compared to a permuted control: in this case, DIOgene leverages gene expression alone.

### 3.2 DIOgene provides a good trade-off between MSE and prior integration, and outperforms state-of-the-art approaches

In order to evaluate the added value of tuning TFBM contributions in a gene-specific manner with DIOgene, we compared the global properties of GRNs optimized by DIOgene to GRNs inferred with a parameter *α* identical for all genes as in previous approaches (Greenfield *et al.* 2013, Petralia *et al.* 2015, Miraldi *et al.* 2019, Skok-Gibbs *et al.* 2022). We used for this weightedLASSO and weightedRF, as well as the methods mLASSO-StARS (Miraldi *et al.* 2019) and iRafNet (Petralia *et al.* 2015) estimated for three global integration strengths *α* (0, 0.5, and 1) (see Supplementary Methods F and G). Additionally, we included an ElasticNet version of weightedLASSO, weightedEN, that bears strong similarities with the existing MEN algorithm (Greenfield *et al.* 2013) (Supplementary Method H). All sparse GRNs were built with a target density of 0.005, resulting in a total of 1432 edges.

**Figure 3.**
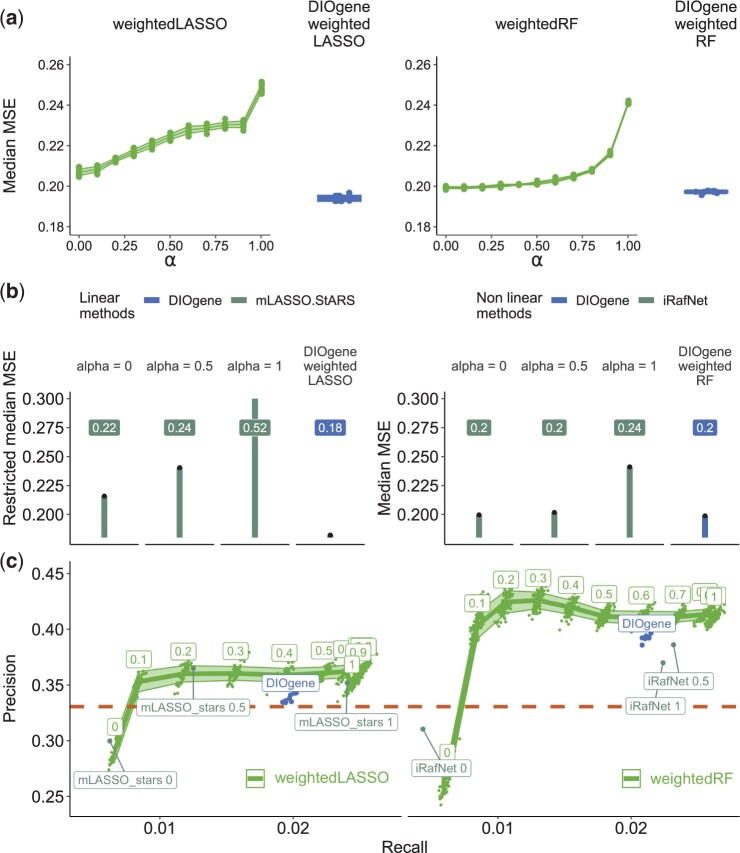
Gene-specific integration of TFBMs to gene expression with DIOgene optimizes model performance and outperforms linear and non-linear state-of-the-art approaches. (a) Median MSE of the nitrate-responsive genes, for weightedLASSO and weightedRF on a resolutive range of global integration strengths *α*, and on DIOgene’s optimization of *α*. (b) Median MSE of the nitrate-responsive genes for mLASSO-StARS (Miraldi *et al.* 2019) and iRafNet (Petralia *et al.* 2015) at three global values of *α* and using DIOgene applied to weightedLASSO or weightedRF. For linear models (left), we use a restricted median MSE, achieved by models learned using only the three most important regulators per target genes. This provides MSE estimates at comparable sparsity levels (see Supplementary Method I). (c) Precision as a function of recall in the inferred GRNs (1432 edges, density = 0.005) against DAP-Seq interactions (O’Malley *et al.* 2016), for a resolutive range of global *α* values and for DIOgene. Precision and recall achieved by existing algorithms, mLASSO-Stars (Miraldi *et al.* 2019) and iRafNet (Petralia *et al.* 2015), are overlaid in darker green for three global values of *α* (0, 0.5, and 1). The precision of the prior TFBMs network of nitrate-responsive genes (31 956 edges, density = 0.32) is shown by the dashed line.

First, the median MSE of GRNs optimized with a global *α* displays a marked increase as the contribution of TFBMs is reinforced in weightedRF and weightedLASSO (Fig. 3a), but also in mLASSO-Stars and iRafNet (Fig. 3b, Supplementary Fig. S5a and b). This is in agreement with the previous observation that, for a significant number of target genes, TFBMs deteriorate model predictions (Fig. 2a). Second, reinforcing TFBMs contribution in GRN models equally for all genes increases both precision and recall against DAP-Seq interactions (Fig. 3c, Supplementary Figs S5c and d and S6a and b). Noteworthily, both weightedLASSO and weightedRF display a strong increase in precision with a global *α*, especially between *α *= 0 and α=0.1, and weightedRF demonstrates a clear advantage over weightedLASSO, with a precision exceeding 0.4. Both models outperform the precision of the prior PWMs network for α>0, indicating that using expression data to choose relevant links from TFBM-supported interactions helps predicting TF binding in an *in vitro* context. Recall values between the linear and non-linear models are similar. Thus, in GRNs inferred by weightedLASSO, weightedRF, and their closest competitors mLASSO-StARS and iRafNet, increasing data integration strength globally improves precision and recall (Fig. 3c) but necessarily comes with a deterioration of model predictions of the target gene expression (Fig. 3a and b).

In contrast, gene-specific optimization of *α* with DIOgene can improve at the same time the prediction of target gene expression and the retrieval of DAP-Seq interactions. In fact, for both the linear and non-linear case, it provides a median MSE lower than any median MSE obtained with a global *α*, for comparable models inferred by weightedLASSO, weightedRF, mLASSO-StARS, and iRafNet (Fig. 3a and b). At the same time, GRNs obtained with DIOgene achieve near-optimal precision and recall, as compared to global *α* curves (Fig. 3c). In this context, we actually argue that it is desirable to tolerate sub-optimal precision and recall results while prioritizing low MSE. In addition to the drawbacks of gold standards presented in Section 1, we observed that precision and recall also increase with *α* in shuffled datasets, where the wrong expression profiles are attributed to the TF (Supplementary Fig. S6a and b, shuffled datasets). This illustrates that precision and recall can be increased simply by enforcing data integration, even when gene expression data is uninformative. We thus think that, in the context of this study and similar ones, these statistics are unfit to properly tune the amount of a complementary omic source to incorporate into GRN inference. In summary, DIOgene improves upon existing algorithms by allowing TFBMs integration to increase the retrieval of binding events, but without degrading the prediction of gene expression.

### 3.3 DIOgene improves upon a naive MSE minimization

Finally, we evaluated the benefit of optimizing the MSE divergence from a shuffled baseline as proposed in DIOgene over a simpler approach that would minimize the MSE directly. First, note that MSE minimization can be an appropriate criterion in cases where integrating prior information reliably improves model accuracy (which is the case here for certain target genes), but this may not always be the case with noisy or incomplete priors. DIOgene and the minimal MSE approach agree on setting α>0 for a large group of genes (793 and 657 for weightedLASSO and weightedRF, respectively). They also both set *α* = 0 for 250 and 571 genes. Thus, our scheme and the minimal MSE approach perform data integration on globally similar sets of target genes. In contrast, some target genes are set to *α* = 0 by DIOgene but not by the minimal MSE (273 and 82): these genes reach a minimal MSE for α>0, but do not diverge sufficiently from the synthetic null hypothesis, and are thus removed from the data integration set by DIOgene (Supplementary Fig. S7a). On the contrary, other target genes are set to α>0 by DIOgene and to *α* = 0 by the minimal MSE (146 and 116). These genes typically display an increasing MSE, but this increase is statistically lower than that of the shuffled control (Fig. 1e). Finally, we focused on the sets of genes for which we specifically integrate TFBMs in one approach but not the other, and computed precision and recall curves of the corresponding sub-networks. At a small and expected MSE cost (Supplementary Fig. S7b), the results showed better precision and recall performance (Supplementary Fig. S7c and d) for the sets of genes considered for data integration by DIOgene as compared to those considered for data integration by the minimal MSE.

### 3.4 DIOgene improves the modeling of nitrate signaling

Finally, we assessed the ability of the inferred GRNs to model nitrate induction pathways in *Arabidopsis* roots by comparing them to state-of-the-art knowledge about this well-documented response (Bellegarde *et al.* 2017, Vidal *et al.* 2020). In order to identify the regulators predicted as important players in nitrate response by our models, we ranked regulators by out-degree in the inferred GRN. This was done for both weightedLASSO and weightedRF, either in GRNs inferred with a global value of *α* = 0, *α* = 1, or with DIOgene’s $\alpha_{t,\text{opt}}$ (Supplementary Fig. S8a). A first observation is that, regardless of the chosen model or data integration strategy, the 25 TFs with highest out-degree contain previously known master regulators of nitrate response. This includes DIV1 (Cheng *et al.* 2021), TGA1 and TGA4 (Alvarez *et al.* 2014), as well as the homologs HHO2 and HHO3, belonging to the NIGT family and identified as repressing the expression of crucial nitrate transport genes (Kiba *et al.* 2018, Safi *et al.* 2021). Interestingly, we also uncover VRN1 and CRF4 as connectivity hubs in all inferred GRNs. These regulators were respectively proposed as candidate and validated actors in nitrate signaling pathways in the studies that generated the transcriptomic data used here (Varala *et al.* 2018, Brooks *et al.* 2019). Overall, whole-GRN measures of gene connectivity showed that genes involved in the regulation of nitrate pathways, nitrate uptake, transport, and metabolism (Supplementary Table S3) have a significantly higher total degree than other genes, in both globally optimized (at *α* = 0 and *α* = 1) and gene-specifically optimized GRNs (Supplementary Fig. S8b).

On another hand, we noticed that gene-specific calibration of data integration uniquely retrieves important regulators of nitrate nutrition that were not present in the 25 most connected TF of the inferred GRNs with a global *α* (*α* = 0 or *α* = 1). In the case of weightedLASSO, only the proposed gene-specific data integration strategy retrieves NLP7, which has been intensively documented as one of the main orchestrator of the early nitrate response (Marchive *et al.* 2013, Alvarez *et al.* 2020). This is also the case of PHL1, a TF involved in the links between nitrate and phosphate signaling *via* NIGT-mediated regulations (Ueda *et al.* 2020). In the case of weightedRF, the proposed gene-specific optimization of data integration enabled the identification of ABF2, a TF recently defined for its role in the endodermal response to nitrate in *Arabidopsis* (Contreras-López *et al.* 2022). It also put forward new TFs as original candidates for nitrate response regulation. This includes HHO6, a member of the NIGT family not yet characterized for its role in the response to nitrate (Kiba *et al.* 2018, Safi *et al.* 2021), but also BZIP53, a TF involved in the regulation of several facets of metabolism (Garg *et al.* 2019). Thus, this analysis reveals that this method of inference, *via* the optimization of data integration in a gene-specific manner, not only recovers the information previously reported in the literature, but also brings to light new factors likely to be involved in this response.

## 4 Discussion

The helpfulness of data integration is very often taken as granted in systems biology. Our work shows that it can in fact have very diverse effects on the modeling of gene expression, and that TFBMs incorporation can be at the expense of model predictive capabilities for a significant number of target genes. We thus propose to replace bulk data integration by a finely tuned hypothesis-driven data integration, calibrated individually for each target gene. Our optimization scheme, DIOgene, leverages TFBMs in a way that their joint use with gene expression improves the target gene expression prediction over a simulated null hypothesis. In our plant biology case study, GRNs inferred with this approach preserve an optimal predictive performance on gene expression, while exhibiting near-optimal precision and recall against DAP-Seq. Such an outcome cannot be obtained through a global, non-specific tuning of *α*, as illustrated by our benchmarks against existing algorithms (Greenfield *et al.* 2013, Petralia *et al.* 2015, Miraldi *et al.* 2019) and our unified re-implementations weightedLASSO and weightedRF. Moreover, such conclusions hold for both the linear and non-linear regression cases, showing some general applicability of our scheme to the most common models in the field. Overall, 567 target genes have $\alpha_{t,\text{opt}}>0$ in both weightedLASSO and weightedRF. This significant intersection indicates that the two models mostly agree on a group of genes for which data integration is beneficial, even though specificities remain (Supplementary Fig. S9a). Although not in the scope of this article, exploring these differences and the structure of the corresponding GRNs would be a great way to test the impact of linearity and parametric assumptions in the modeling of multi-omics GRNs. The reason why some target genes do not benefit from TFBMs integration could stem from various factors, either technical or biological. Mining the consensual lists of genes for which *α *= 0 or α>0 in both models revealed few differences regarding gene expression, function, sequence, and structure characteristics (Supplementary Fig. S9b). Even though certain genes do not take advantage of TFBM data integration, they might in fact benefit from the integration of another form of complementary data. Thus, trying to incorporate several other types of prior data and then comparing the lists of genes not benefiting from these integrated priors could be helpful. Further work would be needed to formulate hypotheses about the potential underlying regulatory mechanisms, and also to assess the role of other forms of regulations like post-transcriptional and post-translational modifications in these results.

Several limitations of this study should be reminded to the reader. First of all, as in all works inferring GRNs from expression data, the expression of the regulators is taken as a proxy for their activity. This assumption is not always valid, which motivated the estimation of TF activities in other studies, typically leveraging motifs or binding experiments combined to gene expression (Li *et al.* 2014, Arrieta-Ortiz *et al.* 2015, Skok-Gibbs *et al.* 2022). Our form of data integration, where TFBM-supported regulators have a stronger contribution in the estimated model, is another way to move away from this limitation. Even though this is a step toward more causality, challenges remain. For instance, strong levels of correlation in the input data are still hindering accurate GRN inference, as a lot of pairs of regulators have correlated expression profiles. When two TFs have a correlated expression profile, TFBM information can be used to select the relevant one. However, TFBM information is not always helpful in this task: sometimes the PWMs of both TFs are unknown, or their PWMs represent roughly the same motif. As a consequence, identifying the meaningful regulator is not always guaranteed. Correlation between variables also impacts the design of simulated null datasets, such as the one we propose, as the simulated null data may sometimes partly resemble the original data only by chance. Bringing more diverse expression profiles into the simulated datasets could be envisioned. The lack of a PWM for a significant number of TFs is also a problem, amplified in non-model organisms. This limitation should be further reduced as PWMs databases are enriched and maintained by the community in the years to come, or as new computational methods are developed to predict binding affinities directly from DNA and protein sequences (Barissi *et al.* 2022). In the TFBMs prior, we can also note the high chance of false positives when scanning PWMs, and the questionable biological relevance of ubiquitous TFBMs with low complexity in the promoters of target genes (Supplementary Fig. S1c). Similarly, non-canonical binding events can be driven by features like DNA shape, structure, or repeat sequences (Samee 2023), that are not directly modeled in our approach. From a computational perspective, the computing time in DIOgene could be reduced in several ways. This includes running specific analyses to estimate the minimal number of repetitions needed to properly assess the MSE and EDI curves, or using a dedicated procedure for pooling genes with similar MSE curves prior to estimating *α*, so that fewer repetitions per genes are needed. Another way to reduce the number repetitions would be to keep identical bootstrap samples between the true data and our simulations of *H*_0_, thus taking advantage of the statistical power offered by paired comparison tests.

In addition to the aforementioned perspectives, the application of the proposed data integration strategy to other complex organisms is a promising lead. In this work, TFBMs influencing gene expression were assumed to be located in the promoter regions of their target genes because very few distal regulations have been reported in *Arabidopsis*, and are still poorly understood (Lin *et al.* 2021). In organisms where regulation by distant enhancers is well documented and responsible for tissue-specificity (Andersson *et al.* 2014, Kamal *et al.* 2023), delineating enhancer regions may be achieved through the use of additional molecular layers such as chromatin accessibility, chromatin contacts, or eQTLs. Enhancers and promoters could then be scanned for TFBMs, linked to their target genes and further guide GRN inference.

In our case study, we favored the use of model prediction performance as a quality metric because it is a condition specific metric available for all target genes and orthogonal to the integrated TFBMs priors, which is often not the case of current experimental gold standards. Our results indicate that instead of directly minimizing prediction error as a function of TFBMs contribution, the comparison to a shuffled baseline improved inferred GRNs (Supplementary Fig. S7). In essence, any inference method where data integration is tuned by a parameter could be optimized based on such a simulated null dataset. As a general guideline, we believe that both the monitored quality metric and the simulated baseline should be carefully designed in order to test a clear and relevant hypothesis for the problem at hand. Even more generally, the concept of synthetic null datasets for *in silico* negative controls is gaining interest in genomic analyses. For example, scDEED (Xia *et al.* 2024), clusterDE (Song *et al.* 2023a), or scDesign3 (Song *et al.* 2023b), are examples of procedures enhancing statistical pipelines for single-cell data analysis via the use of randomized null hypotheses, which are likely to enhance rigor and causal discoveries in the field.

## Supplementary Material

btae415_Supplementary_Data

## Data Availability

The RNA-Seq data for the response to nitrate induction were downloaded from the GEO accession GSE97500. The PWMs used to build the TFBM dataset were retrieved from JASPAR and the Plant Cistrome Database. To identify Arabidopsis TSSs and promoter regions, we relied on the TAIR10 GFF3 file. The regulators of Arabidopsis used for GRN inference are the union between PlnTFDB and AtTFDB. All results can be reproduced with the code available in the GitHub repository: https://github.com/OceaneCsn/integrative_GRN_N_induction.
