# Supplementary material for: Optimizing data integration improves gene regulatory network inference in Arabidopsis thaliana
Source: Bioinformatics. 2024 Jun 24;40(7):btae415. doi: 10.1093/bioinformatics/btae415 (PMC11227367; doi:10.1093/bioinformatics/btae415)
Supplement: btae415_Supplementary_Data [file btae415_supplementary_data.zip › Supplementary_Materials.pdf]

Supplementary Methods

A. Experimental data analysis of the Arabidopsis dataset

Gene expression was measured in seedling roots at times 0, 5, 20, 30, 45, 60, 90, and 120 minutes after nitrate or control treatments (N = 45 samples) (1). Although samples at 10 and 15 min were also available, we discarded them, as they were clear outliers in the first dimension of a Principal Component Analysis suggesting a batch effect (Figure S1a). The RNA-Seq raw counts were normalized via the TMM method (2), and lowly expressed genes were removed. We selected differentially expressed genes responding to nitrate induction in time by testing the interaction terms between nitrate treatment and time modelled as natural splines, as proposed in the original article that produced this dataset (3; 1).

We define a promoter sequence in Arabidopsis as the sequence spanning -1,000 bp and +200 bp around gene start as defined in TAIR10, because this interval has been estimated to contain 86% of binding sites in plants (4). TFBM information, encoded by Position Weight Matrices (PWM), was retrieved from the JASPAR (5) and The Plant Cistrome (6) databases. Among the 201 nitrate-responsive regulators, 70 regulators were associated with a known PWM in the union of these databases. FIMO (7) was used to find occurrences of these 70 PWMs with a significance threshold of $1\mathrm{e}^{-4}$ in promoter sequences. When several occurrences were identified in one promoter, the maximum score was kept. The TFBM occurrences within nitrate-responsive gene promoters form a prior TFBM network between TFs and targets in which a target promoter harbors in average 23 TFBM (Figure S1b) and a given TFBM is found in approximately 500 target promoters in average (Figure S1c).

B. Generalized Linear Model with Weighted LASSO (weightedLASSO)

We implemented weightedLASSO and weightedRF with the intent to represent current algorithms performing integrative regression-based GRN inference in a unified manner. They are generic re-implementations of available methods under the same programming language, giving access to internal regressions error estimates, with unified inputs and the presence of an additional parameter α controlling data integration strength. This has the advantage of facilitating reproducible model comparisons and downstream efforts to optimize data integration strength for both models.

As RNA-Seq experiments generate count data, we model the expression of a target gene t in the condition i as a Poisson-distributed variable $Y_{t,i} \sim \mathcal{P}(\mu_{t,i})$. The parameter of the Poisson distribution $\mu_{t,i}$ is estimated on the log scale as a linear combination of the expression values of the regulator genes, with $x_{r,i}$ the expression level of regulator r in condition i:

$$\ln(\mu_{t,i}) = \beta_{t,0} + \Sigma_{r=1}^{R} \beta_{t,r} x_{r,i} \tag{1}$$

We employ a LASSO penalty in order to overcome the high-dimensional setting and to select the most predictive regulators. In addition, we propose to use differential shrinkage in order to favor the selection of TFBM-supported variables. Differential shrinkage for the LASSO (weighted LASSO) allows to modulate the penalty strength of each variable individually, in a way that regulators with a binding motif in the target's promoter are less penalized during model adjustment. We model differential shrinkage using specific penalty coefficients $w_{t,r} \in [0,1]$ defined as a linear function of the TFBM prior $\Pi_{t,r}$ and α (Figure S2b):

$$w_{t,r}^{LASSO} = 1 - \Pi_{t,r}\alpha \tag{2}$$

For each target gene t, the function to minimize for model estimation is thus:

$$\mathrm{argmin}_{\beta_t} \left\{ -\log\mathcal{L}(\beta_t; X, Y_t) + \lambda \Sigma_{r=1}^{R} w_{t,r}^{LASSO} |\beta_{t,r}| \right\}, \tag{3}$$

where

λ controls the overall strength of the penalty,

R is the total number of regulator genes,

N is the total number of expression measurements for t

$\log\mathcal{L}(\beta_t; X, Y_t)$ is the log-likelihood function.

The value of λ is learned from 5-fold cross validation. We relied on the `glmnet` (8) implementation of the LASSO, with the `penalty.factor` argument specifying differential shrinkage weights. In practice, for genes with a number of TFBM-supported regulators exceeding the total number of experiments considered, the model cannot be estimated at $\alpha = 1$. We thus set α to $1 - \epsilon$ instead of 1, with $\epsilon = 10^{-4}$.

To further reduce over-fitting problems and improve robustness, we also included a bootstrap procedure to the LASSO (9; 10; 11), as already used in some GRN inference approaches using a linear model (12; 13). Hence, instead of fitting a single generalized linear model, S models are adjusted on a bootstrapped version of the data as follows:

1. N observations are sampled with replacement from the N available experimental conditions (bootstrapping).
2. The N bootstrapped observations are randomly partitioned into 5-cross validation folds. We ensure that duplicated observations during bootstrapping are grouped within the same fold.
3. A model is fitted by minimizing Equation (3) during cross-validation, allowing to learn the value of the sparsity, λ_{1se}. λ_{1se} is the largest value of λ in the λ grid less than one standard deviation away from the value of λ minimising prediction error on the cross-validation test folds.

In this study, results are presented for $S = 50$.

C. Weighted Random Forests (weightedRF)

Non-linear regressors such as ensembles of regression trees model combinatorics of regulators and complex relations between a target gene and the expression of its regulators. As inspired by iRafNet (14), we model data integration in RF by increasing the use of regulators supported by a binding motif in the decision nodes of the regression trees. A weightedRF is inferred for each target gene t. At each decision node, the most discriminating regulator is chosen among a subset of $\sqrt{R}$ regulators. This subset, traditionally equiprobably sampled from all the regulators, is submitted here to a weighted sampling where the weights encode prior knowledge about the regulators, growing with data integration strength α and prior value $\Pi_{r,t}$. Regulators with a high prior value are more likely to get chosen among the $\sqrt{R}$ regulators tested to create a decision node. More formally, we define that the chance of a regulator r to get picked in the decision node for the target gene t is proportional to the weight $w_{r,t}^{RF}$ (Figure S2c):

$$w_{r,t}^{RF} = \begin{cases} -\sqrt{1-(\alpha-1)^2}+1 & \text{if } \Pi_{r,t} = 0 \\ 1-\alpha & \text{if } \Pi_{r,t} = \frac{1}{2} \\ \sqrt{1-(\alpha-1)^2}+1 & \text{if } \Pi_{r,t} = 1 \end{cases} \tag{4}$$

In practice, these functions allow that in both cases, when $\alpha = 1$, variable selection is performed among regulators with $\Pi_{rt} = 1$

only (Figure S4). One weighted tree is learned for each of S bootstrapped samples. In this study, results are presented for $S = 2000$. The S trees are then aggregated into one RF per target gene. We implemented the new weight function upon the iRafNet R and C++ code, along with the possibility to restrict variables to regulators in regressions, which was not permitted initially, and with the addition of a new feature importance metric (Equation 7).

D. Error in predicting gene expression (MSE)

We assess the ability of weightedRF and weightedLASSO to accurately predict gene expression via the Mean Squared Error (MSE) metric. It is measured on Out Of Bag conditions (OOB) that were left out of the bootstrapped samples (and consequently not used in weightedRF and weightedLASSO models training). For weightedLASSO, the MSE for a target gene t, and a given α is

$$MSE_{t\alpha} = \frac{1}{S}\Sigma_{m=1}^{S}\frac{1}{N_{OOB_m}}\Sigma_{i\in OOB_m}(y_{ti\alpha} - \hat{y}_{mti\alpha})^2 \quad (5)$$

where

m corresponds to one of the S bootstrapped LASSO models,
OOB_m refers to all conditions i that are OOB for m,
N_{OOB_m} refers to the cardinal of OOB_m,
$\hat{y}_{mti\alpha}$ is the prediction of m on the condition $i \in N_{OOB_m}$.

For weightedRF, the MSE for a target gene t and α is

$$MSE_{t\alpha} = \frac{1}{N}\Sigma_{i=1}^{N}\frac{1}{S_{OOB_i}}\Sigma_{m\in OOB_i}(y_{ti\alpha} - \hat{y}_{mti\alpha})^2 \quad (6)$$

where

i corresponds to one of the N experimental conditions,
OOB_i refers to all trees m for which i is OOB,
S_{OOB_i} refers to the cardinal of OOB_i,
$\hat{y}_{mti\alpha}$ is the prediction of $m \in S_{OOB_i}$ on the condition i.

In order to provide MSE values comparable between the different target genes, the MSE is normalized by the variance of the target gene in all figures and results of this study.

E. Importance of a regulator

Previous RF-based models (15), including iRafNet (14) which inspired weightedRF, used the traditional node purity metric. However, it is tailored for tree-based approaches only, and was shown to be not suitable to interpret variable importance in RFs in the presence of dependencies and interactions (16; 17). We therefore define a common importance metric for both for weightedLASSO and weightedRF. This importance measure between a regulator r and its target t is given by the relative increase of MSE measured on the OOB (Equation 5 and 6), induced by shuffling the expression values of r when making the prediction $\hat{y}_{mti\alpha}$:

$$\text{Importance}_{rt\alpha} = \frac{MSE_{t\alpha,\text{shuffle}(r)} - MSE_{t\alpha}}{MSE_{t\alpha,\text{shuffle}(r)}}. \quad (7)$$

This metric is very closely related to the original "mean decrease accuracy" approach proposed by Friedman (18). The only difference is we normalize the MSE difference by $MSE_{t\alpha,\text{shuffle}(r)}$ to ensure that this statistic is comparable between different target genes and is included in the $[0,1]$ interval as similarly proposed in previous studies (19; 20).

F. Comparison to mLASSO-StARS

We benchmark weightedLASSO against its closest and most recent competitor, mLASSO-StARS (13). This method was initially implemented in Matlab. A python adaptation was integrated into the Inferelator3 (20). However, the python adaptation in the Inferelator3 does not allow to modulate the LASSO penalty strength depending on prior data (data integration is only performed through the upstream estimation of TF activities). In order to circumvent this limitation while relying on open software only, we provide an R implementation of mLASSO-StARS, following the method presented in (13). The following table summarizes the differences between weightedLASSO and mLASSO-StARS.

	mLASSO-StARS	weightedLASSO
Data transformation	log-transformed	None
Generalized linear model family	Gaussian	Poisson
Model sparsity choice	StARS (lasso.stars function (11))	Cross validation (cv.glmnet function)
Importance metric	Selection frequency and partial correlation (Eq. 4 in (13))	Normalized MSE increase (section E)

The R implementation of mLASSO-StARS is available in the project's github `https://github.com/OceaneCsn/integrative_GRN_N_induction/tree/master`. mLASSO-StARS was run with the same variability threshold (0.05) as in (13).

G. Comparison to iRafNet

We benchmark weightedRF against its closest and most recent competitor, iRafNet (14). Because weightedRF directly relies upon the iRafNet code, we simply ran the code with the original modelling choices of iRafNet. The differences are summarized in the following table:

	iRafNet	weightedRF
Data transformation	Scaled (z-score)	None
Importance metric	Node purity	Normalized MSE increase (section E)

H. Comparison to weightedEN and MEN

We implemented an option to turn weightedLASSO into an ElasticNet version: weightedEN. The only difference between weightedEN and weightedLASSO is the ElasticNet tuning parameter of the regularized generalized linear model, that was set to 0.1 in cv.glmnet() instead of 1. The value of 0.1 was chosen in weightedEN because it allowed to obtain a MSE a small as the MSE of mLASSO-StARS, while still performing feature selection.

WeightedEN is very close to the algorithm MEN (19). The differences are that in MEN, the set of possible regulators is reduced in an upstream mutual information analysis, and that the ElasticNet mixing parameter in optimized via a grid search along with λ during cross validation.

	weightedEN	weightedLASSO	MEN
ElasticNet parameter	0.1	1	CV grid search

I. MSE differences between weightedLASSO, weightedEN, and mLASSO-StARS

Our experiments showed that weightedLASSO and mLASSO-StARS differ in terms of median MSE (Figure S10a). mLASSO-StARS has a lower MSE for $\alpha < 1$, but a very high one at $\alpha = 1$ as compared to weightedLASSO. We hypothesized that the lower MSE of mLASSO-StARS at $\alpha < 1$ was due to different sparsity levels between the LASSO penalty chosen by cross validation and the StARS approach (Figure S10b). Similarly, the Elastic-Net version of weightedLASSO (weightedEN) that also selects more variables than weightedLASSO, produced a smaller MSE, supporting the hypothesis of larger models reducing prediction error. However, when used for GRN inference, large regression models are always pruned to the few most important variables (See the "GRN contruction and evaluation" Methods section). As a frame of reference, the following table summarizes the average number of regulators per target gene for three biologically relevant network densities:

Density	Number of edges	Average number of regulators per target gene
0.005	1432	1.004
0.01	2864	2.008
0.05	14322	10.04

We thus chose the intermediate value of 3 regulators per target gene to recompute the MSE of all the linear models (weightedLASSO, weightedEN, mLASSO-StARS). This MSE is the MSE restricted to the three most important regulators per gene (Supplementary Methods E), which ensures a comparable sparsity level. With this restricted MSE, weightedLASSO provides a similar or smaller MSE than other methods for all α values (Figures 3b and S5b). We also note that weightedLASSO has a smaller MSE in the version restricted to the top three variables than with all variables available, suggesting that it can be prone to overfitting in the context of this study ($N = 45$ and 201 variables).

Supplementary Tables and Figures

Table S1 : Normalized gene expression of the 1426 nitrate-responsive genes in the different treatments and time points of the experiment (1). C: control. N: nitrate induction treatment. Numbers represent time after treatment in minutes.

Table S2 : Gene identifier (AGI) of the 201 nitrate-responsive regulators.

Table S3 : 56 genes involved in the uptake, transport, metabolism and signalling (positive or negative) of nitrate in the roots of Arabidopsis thaliana. Their AGI, gene name and short description are shown. This list of nitrate-related genes was compiled from the literature (21; 22; 23; 24; 25; 26; 1; 27; 28; 29; 30; 26; 31; 32).

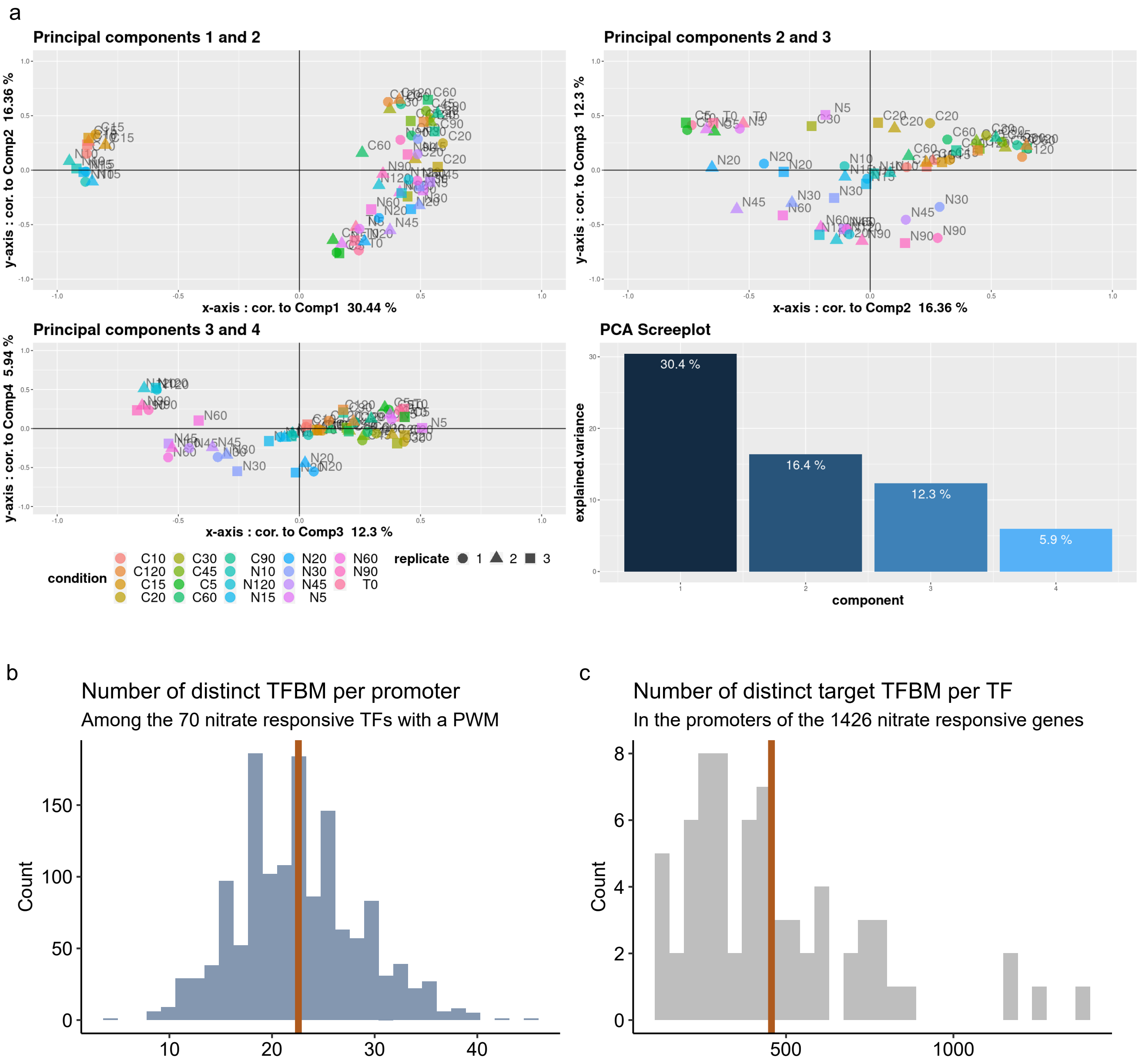

Fig. S1. Description of the Arabidopsis dataset used as a case study.
a. PCA plot of the normalized counts from the RNA-Seq experiment of the dynamic root response to a nitrate treatment (1). The three first subplots show the correlation of each sample to the first 4 principal components. Each color represents an experimental condition made of 3 replicates. C: control. N: nitrate treatment. Numbers represent time in minutes. The first component highlights the samples at 10 and 15 minutes as different from the rest of the experiment, possibly due to a batch effect. The screeplot represents the percentage of variance explained by the 4 first principal components.
b-c: Prior TFBM network of nitrate-responsive genes (1426 promoters and 70 regulators). **b.** Distributions of the number of distinct PWM hits per promoter. **c.** Distribution of the number of distinct promoter hits per PWM restricted to nitrate-responsive genes. The average value of each distribution is represented by the orange vertical line.

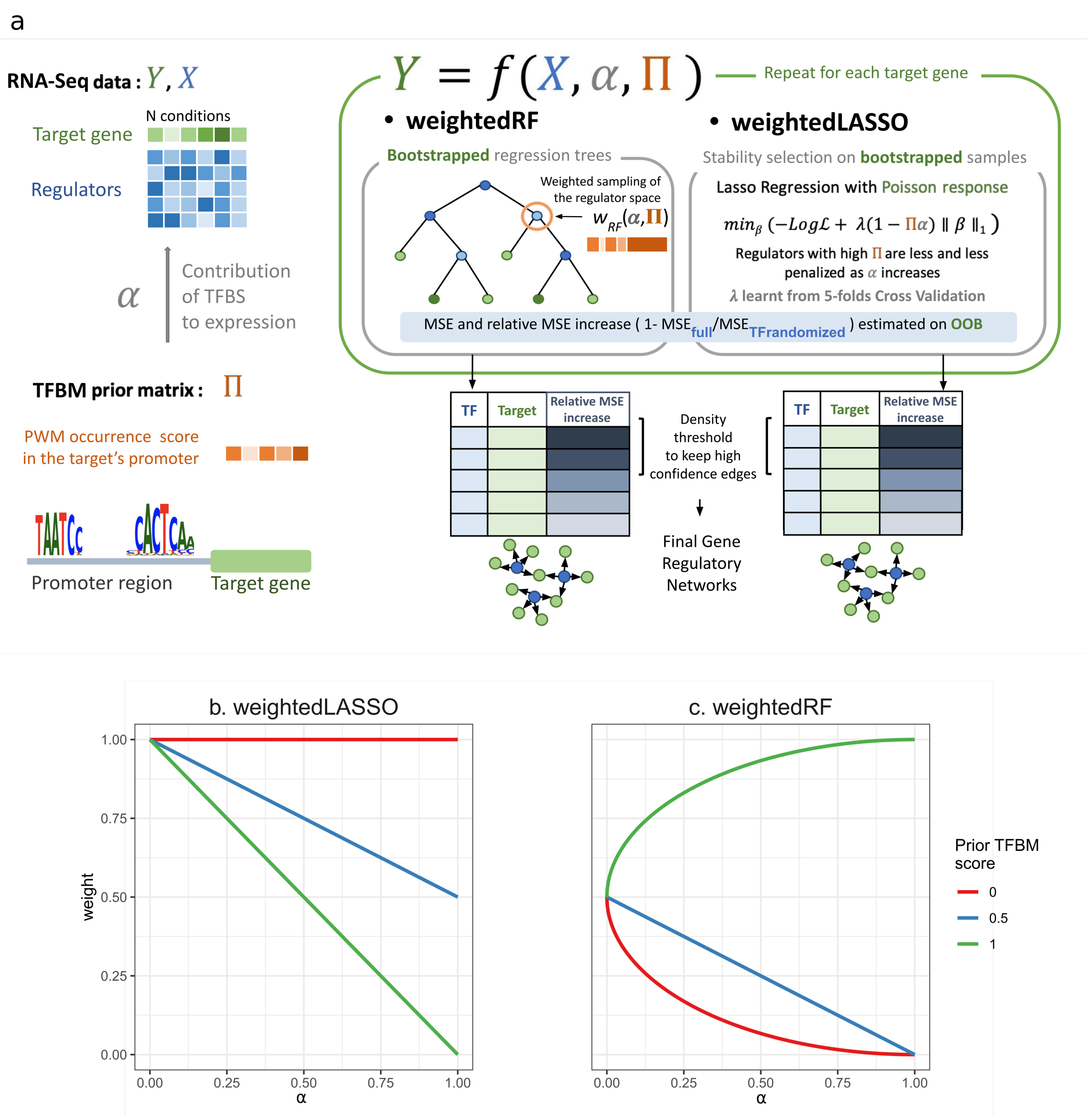

Fig. S2. Overview of weightedRF and weightedLASSO integrative GRN inference procedures.
a. Network inference pipeline. Input data for a given target gene is the vector of expression of this gene Y, an expression matrix for the regulators X, and a TFBM scoring matrix Π. Π contains information about the presence or absence of the regulators PWM in a target gene's promoter. α is a parameter controlling the force of TFBM integration to expression. For each target gene, a regression model is fit to Y with weightedRF or weightedLASSO using X as predictive variables, and favoring the attribution of high influences to regulators with high values in Π. For weightedRF, this prioritization is executed by a weighted subsampling of the regulators space when elongating the regression trees, while it is achieved by differential shrinkage combined to stability selection is weightedLASSO. Once all regulator-gene pairs have been ranked based on their influence in the regression models, final GRN are built by selecting the number of strongest interactions providing a desired network density.
b-c: Functions linking prior TFBM scores (Π_{rt}) to regulator-specific weights during model estimation, depending on integration strength α. b. In weightedLASSO, the penalty strength of the LASSO decreases with α when $\Pi_{rt} > 0$ (Equation 2). **c.** In weightedRF, the sampling weight at regression tree nodes increases with α when $\Pi_{rt} = 1$, and decreases otherwise (Equation 4). Weights are normalized between 0 and 1. In practice, these functions allow that in both cases, when $\alpha = 1$, variable selection is performed among regulators with $\Pi_{rt} = 1$ only (Figure S4).

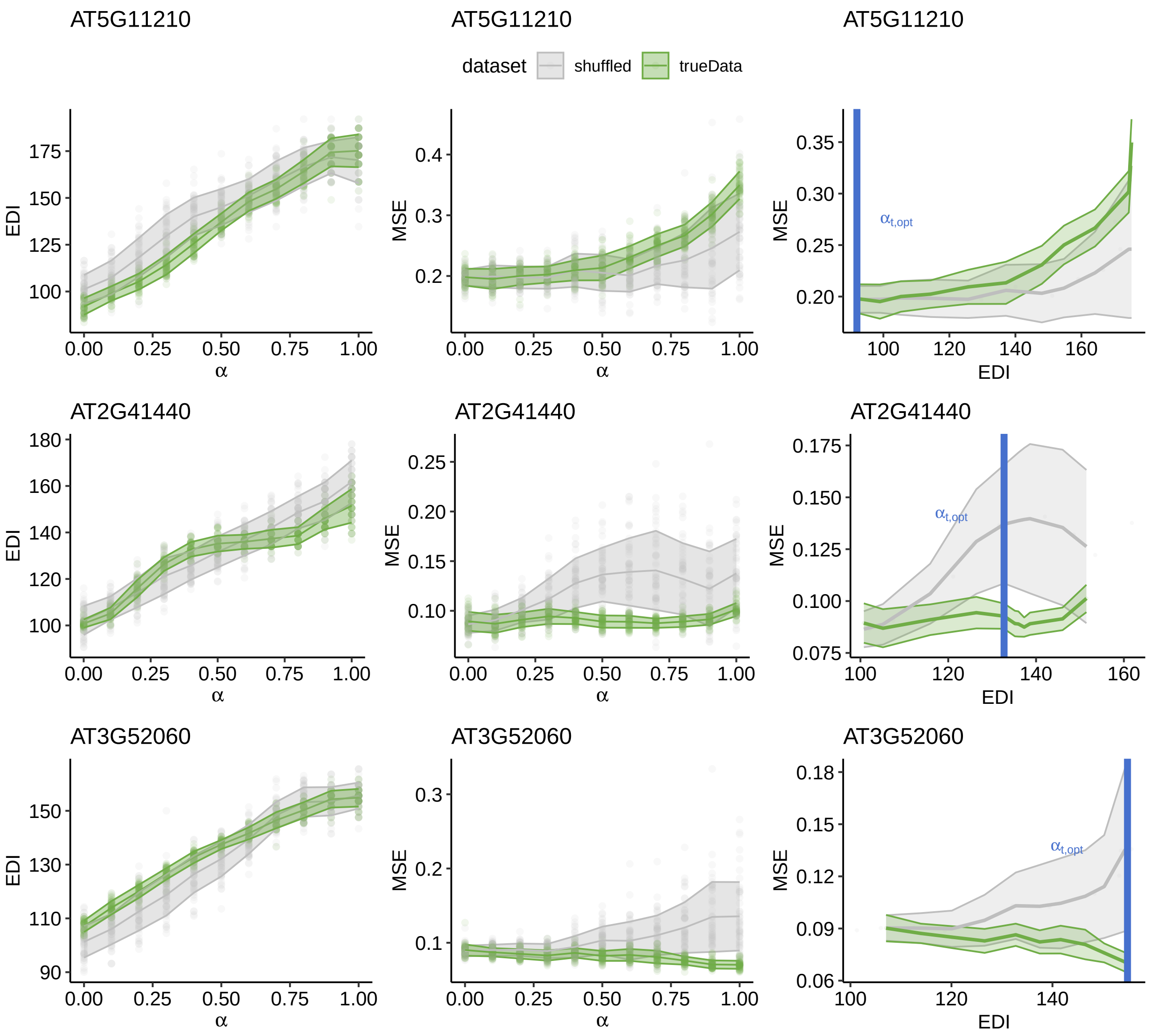

Fig. S3. Different scenarios of data integration with DIOgene in weightedLASSO. For three gene examples in rows, the subplots show the EDI depending on α, the MSE depending on α and MSE depending on EDI for all possible values of α on true data (green) and shuffled null datasets (grey). The MSE is normalized by the variance of the target gene expression. For each value of α, 50 models were estimated and one standard deviation around the mean is represented. The proposed gene-specific α_{opt} (represented as a vertical blue line) is the value for which the MSE is most reduced as compared to the shuffled baseline.

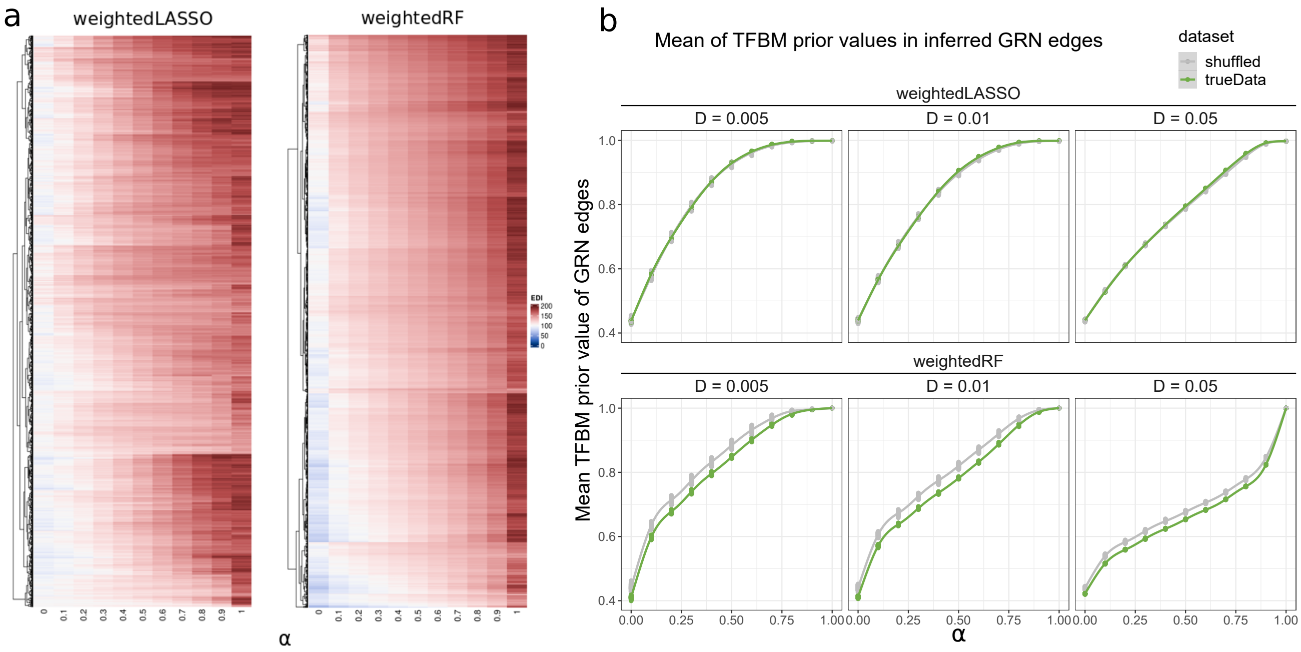

Fig. S4. WeightedLASSO and weightedRF effectively integrate prior knowledge when increasing α.
a. EDI for the 1426 nitrate-responsive genes as a function of α. Values are averaged across 100 replicates for weightedRF and 50 for weightedLASSO. **b. TFBM support of inferred GRN**. TFBM support is the average prior value from the TFBM matrix Π of the inferred TF-target edges. It is shown for three network densities : 0.005 (1432 edges), 0.01 (2864 edges) and 0.05 (14322 edges). A TFBM of 1 means that a GRN is composed only of edges supported by a TFBM. At maximal TFBM integration intensity ($\alpha = 1$), GRN on both true and shuffled data achieve a TFBM support of 1.

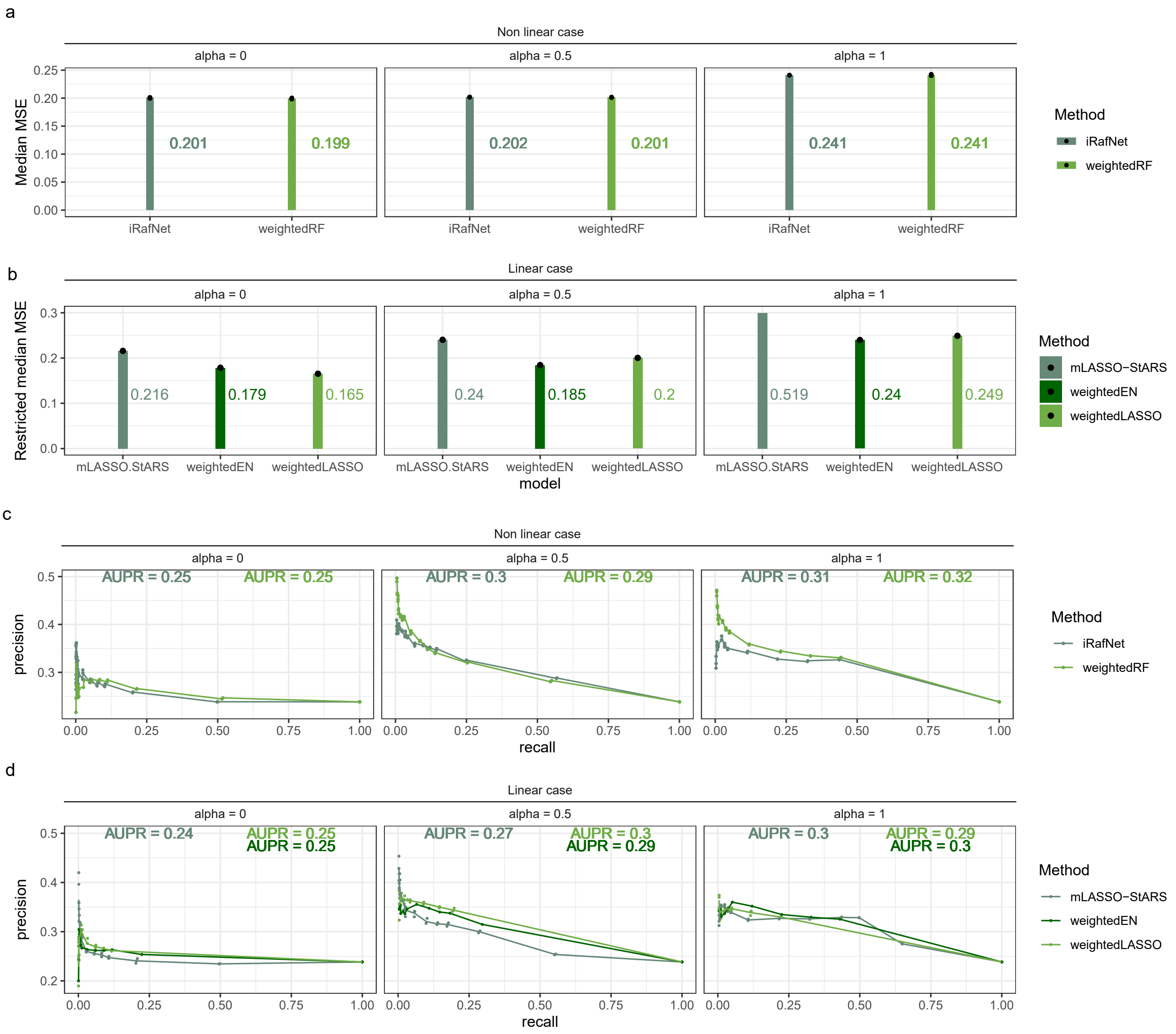

Fig. S5. MSE, and precision/recall of GRNs inferred by weightedRF, weightedLASSO, weightedEN, mLASSO-StARS and iRafNet using three global values of α. See Supplementary Methods F, G, H and I for details about the different algorithms.
a. Median MSE, computed over all nitrate-responsive genes for weightedRF and iRafNet. The MSE is normalized by the variance of the target gene expression. **b**. Restricted median MSE, computed over all nitrate-responsive genes for weightedLASSO, weightedEN and mLASSO-StARS. The restricted MSE is obtained for sparse models learned using only the three most important regulators for each target gene (see Supplementary Methods I). The MSE is normalized by the variance of the target gene expression. The y-axis is truncated so that MSE differences between methods remain visible, otherwise masked by the elevated MSE of mLASSO-StARS at $\alpha = 1$. **c**. Precision and recall curves for weightedRF and iRafNet against DAP-Seq interactions for density thresholds ranging from 0.001 to 1.**c**. Precision and recall curves for weightedLASSO, weightedEN and mLASSO-StARS against DAP-Seq interactions for density thresholds ranging from 0.001 to 1.

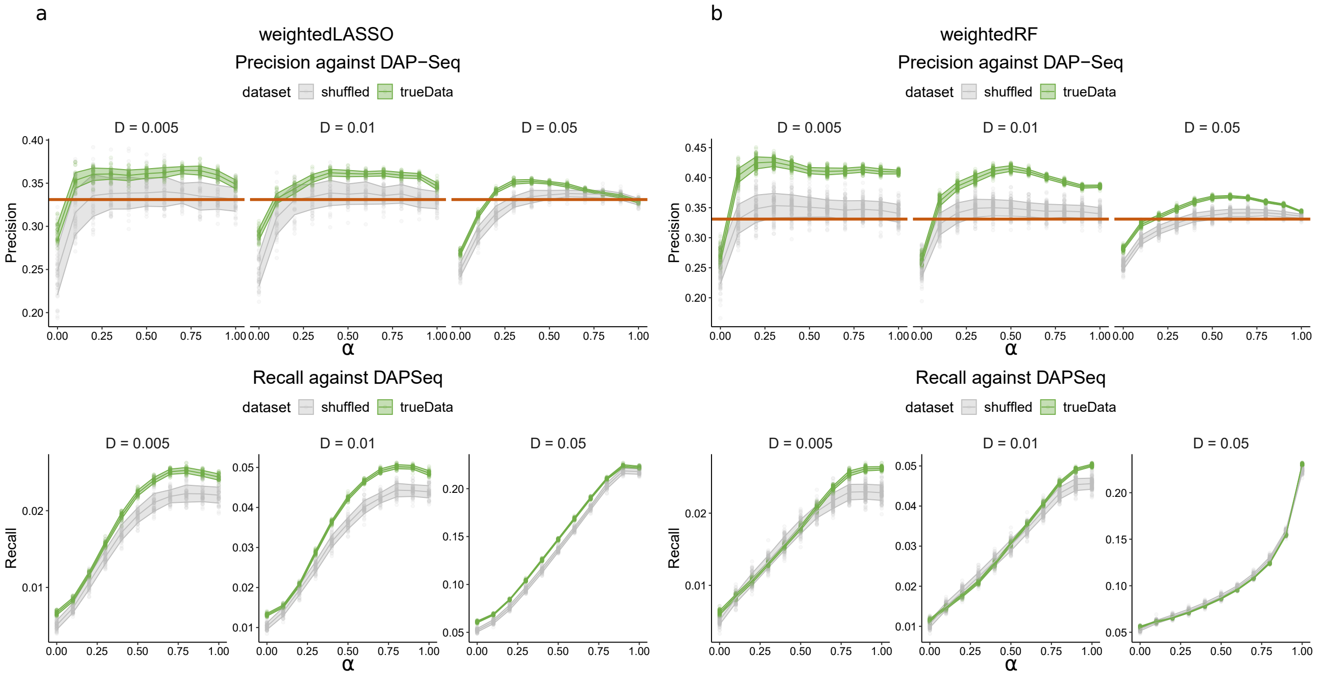

Fig. S6. Precision and recall as a function of α against a DAP-Seq gold standard in weightedLASSO (a) and in weightedRF (b). Precision and recall are show on true data (green) and shuffled null datasets (grey) for three network densities : 0.005 (1432 edges), 0.01 (2864 edges) and 0.05 (14322 edges). Note that datasets representing H_0 are obtained by shuffling the expression profiles of the TFs instead of the TFBM prior values, which has the advantage to keep the TFBM prior consistent with the gold-standard dataset. The precision of the prior TFBM network of nitrate responsive genes (31956 edges, density $= 0.32$) is overlaid in orange.

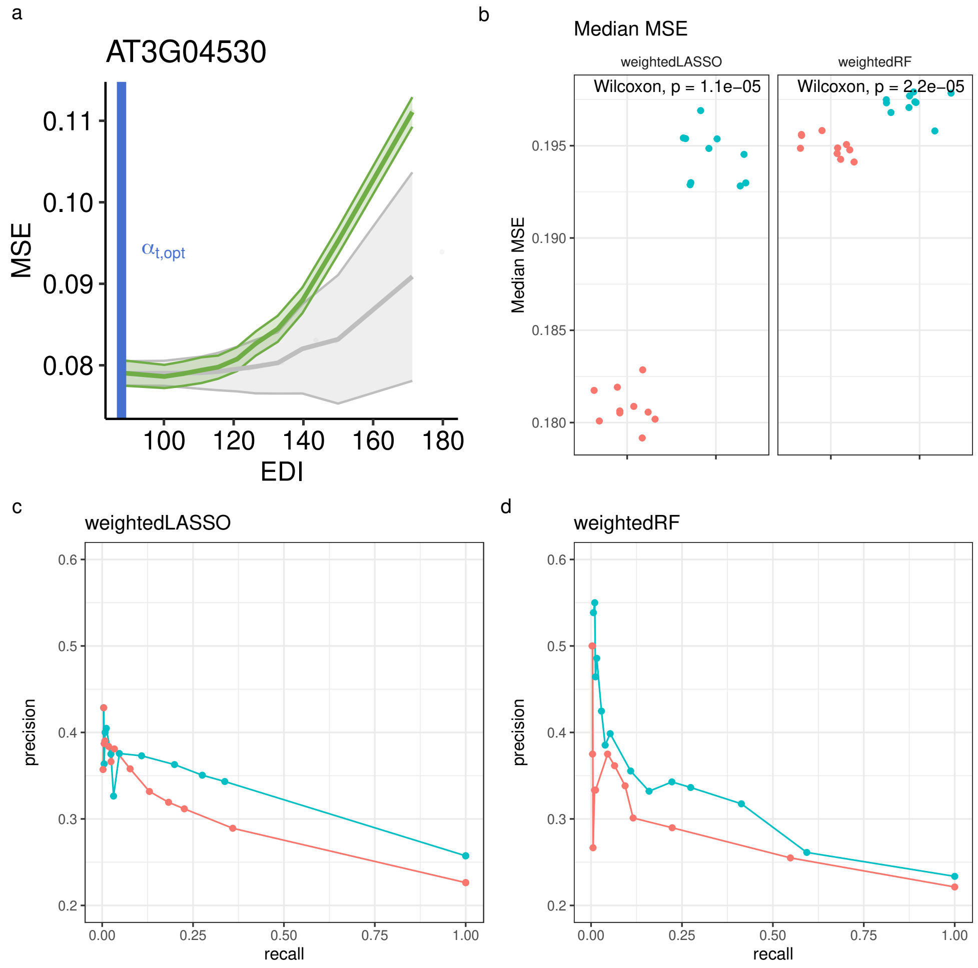

Fig. S7. DIOgene outperforms the minimal MSE approach.

a. Example of a gene in weightedRF for which DIOgene sets $\alpha_{t,opt} = 0$, but the minimal MSE approach would set $\alpha_{t,opt} > 0$. The graph shows the MSE depending on EDI for all values of α on true data (green) and shuffled null datasets (grey). The MSE is normalized by the variance of the target gene expression.

b. Median MSE for DIOgene (blue) and the minimal MSE (red), computed over all nitrate-responsive genes. The MSE is normalized by the variance of the target gene expression. Each dot is a replicate of GRN inference, randomness stemming from different bootstrap sampling.

c-d. Precision and recall curves for densities ranging from 0.001 to 1, computed on sub-GRN made of the edges concerning only target genes for which we integrate TFBM exclusively in one of the compared methods. In red, the genes for which the minimal MSE sets $\alpha_{t,opt} > 0$ but not DIOgene. In blue, the genes for which DIOgene sets $\alpha_{t,opt} > 0$ but not the minimal MSE approach.

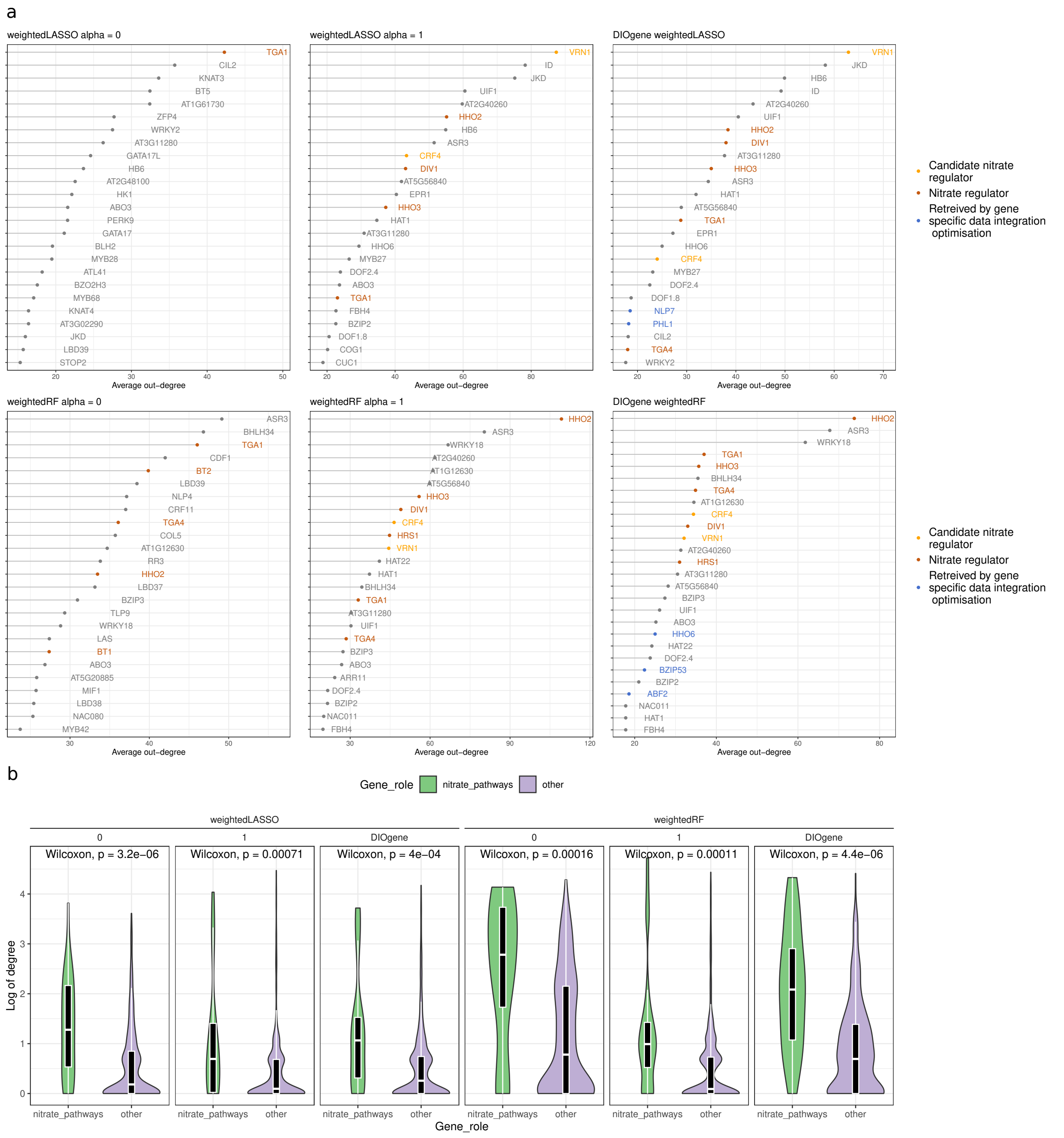

Fig. S8. Nitrate signalling is accurately modeled in GRN inferred with DIOgene.

a. Top connected regulators depending on the model and TFBM integration strategy. In inferred GRN of a 0.005 density, regulators were ranked by their out-degree (number of regulated target genes or other regulators) for weightedLASSO and weightedRF, and for a global value of α (0 or 1), or using the proposed gene-specific approach (DIOgene). Out-degree is averaged across the different runs of the same method to account for inherent stochasticity, and the 25 TFs with the highest out-degree are shown. Regulators already identified in previous studies as important (orange) or candidate (yellow) actors of nitrate signalling and regulation are highlighted. TFs uniquely retrieved by the proposed gene-specific approach as compared to a global optimization for a given model are reported in blue.

b. The overall degree (in-degree and out-degree) of nitrate-related genes is higher as compared to other genes. In inferred GRN (D = 0.005), the total degree of genes is shown for a global value of α (0 or 1), or using DIOgene (specific). Total degree is reported on the log scale. Only genes with at least one connection in the inferred GRN were considered. The list of nitrate-related genes (Table S3) was compiled from the literature (21; 22; 23; 24; 25; 26; 1; 27; 28; 29; 30; 26; 31; 32).

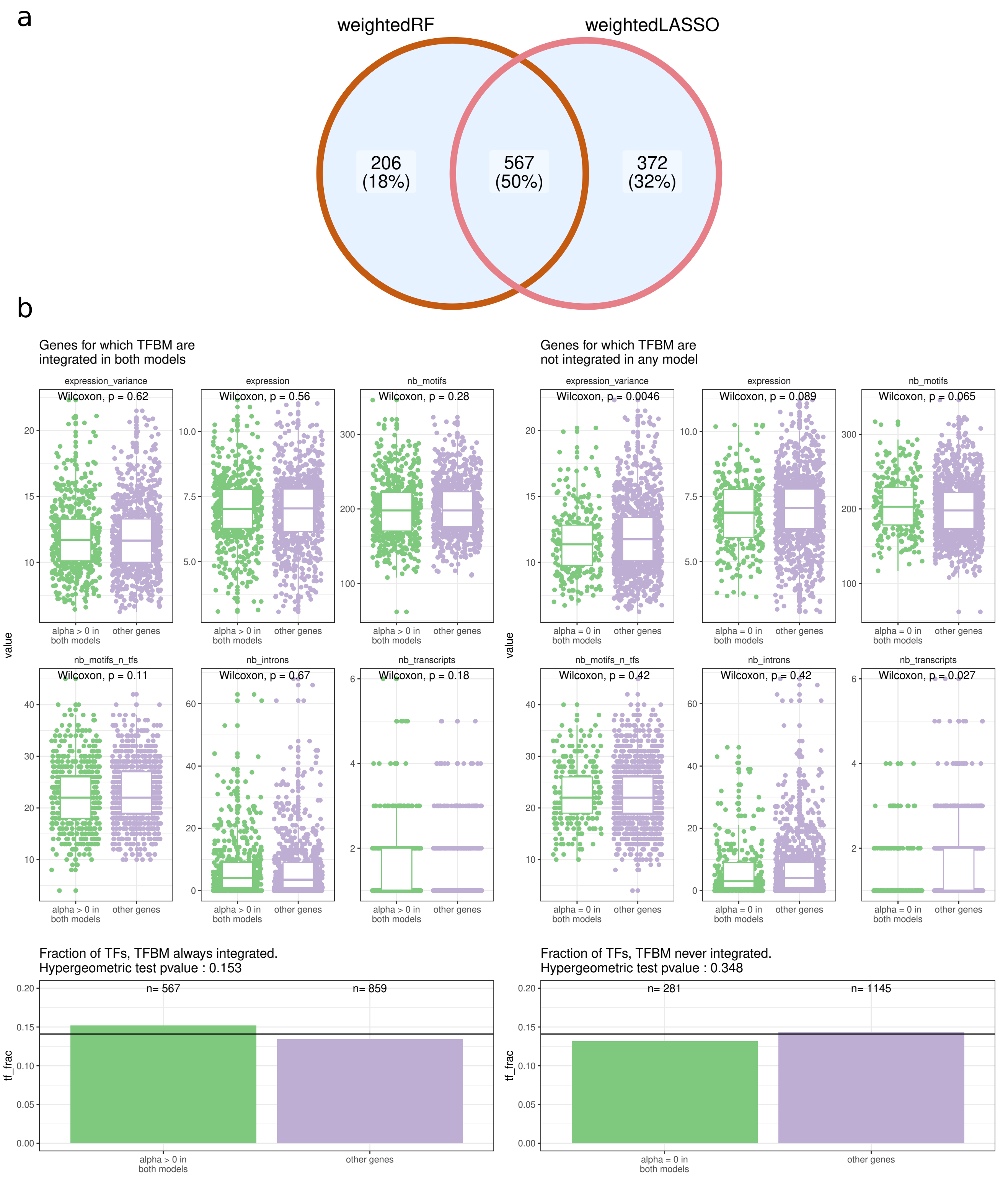

Fig. S9. Gene characteristics depending on their data integration status with DIOgene.
a. Intersection between the genes with $\alpha_{t,opt} > 0$ in weightedLASSO and weightedRF with DIOgene. Enrichment pvalue : $2.7e^{-11}$ (One-sided hypergoemetric test).
b. Functional comparison between genes for which TFBM are consensually integrated ($\alpha_{t,opt} > 0$, left, $N = 567$) or not integrated ($\alpha_{t,opt} = 0$, right, $N = 281$) by DIOgene in weightedRF and weightedLASSO. Expression and expression variance are reported on the log scale. The other characteristics are the number of motifs in the target gene promoter region, either among all known PWM in Arabidopsis (nb_motifs), or only in nitrate responsive regulators (nb_motifs_n_tfs), the number of introns (nb_introns), the number of transcripts (nb_transcripts). The two latter features were retrieved from the TAIR10 GFF annotation. On the last row, proportions of TFs in each group of genes are shown with their enrichment p-value (one-sided hypergeometric test).

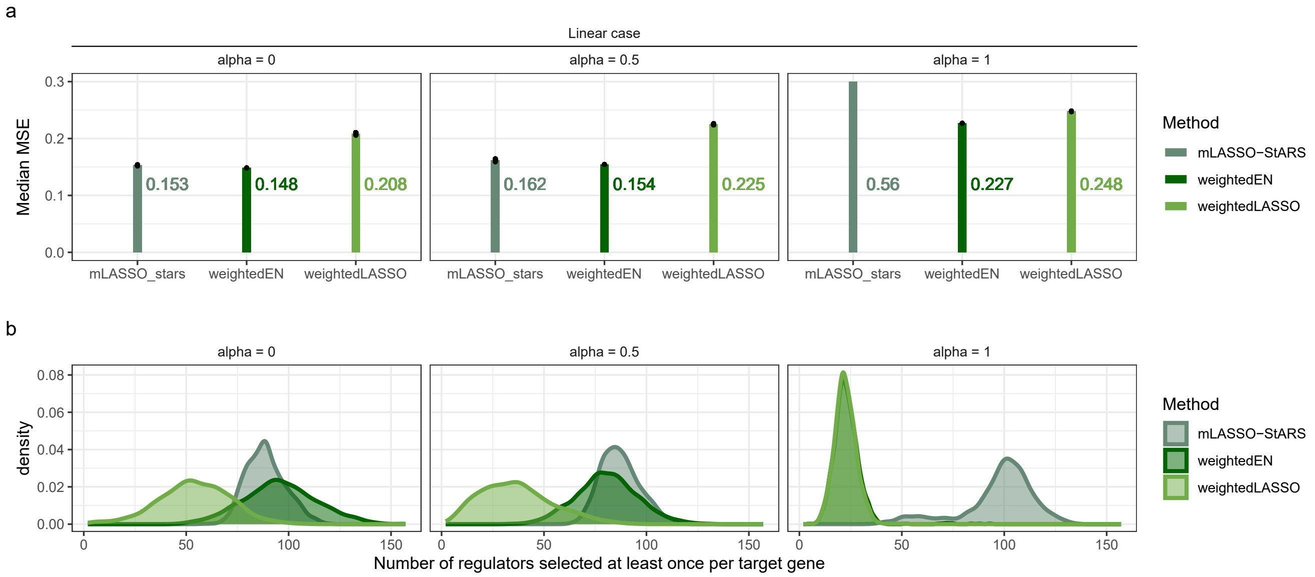

Fig. S10. MSE and number of variables in linear models. See Supplementary Methods F, H and I for details about the different algorithms. **a.** Median MSE (of the full model, unlike in Figure S5b), computed over all nitrate-responsive genes for weightedLASSO, weightedEN and mLASSO-StARS. The MSE is normalized by the variance of the target gene expression. The y-axis is truncated so that MSE differences between methods remain visible, otherwise masked by the elevated MSE of mLASSO-StARS at $\alpha = 1$. **b.** Model sparsity estimates for weightedLASSO, weightedEN and mLASSO-StARS. Mean number of regulators selected at least one time in the stability selection runs of the linear methods.

References

1. K. Varala et al. Temporal transcriptional logic of dynamic regulatory networks underlying nitrogen signaling and use in plants. *Proceedings of the National Academy of Sciences*, 115(25):6494–6499, may 2018.
2. M. D. Robinson and A. Oshlack. A scaling normalization method for differential expression analysis of RNA-seq data. *Genome Biology*, 11(3):R25, 2010.
3. M. E. Ritchie et al. limma powers differential expression analyses for RNA-sequencing and microarray studies. *Nucleic Acids Research*, 43(7):e47–e47, January 2015.
4. C.-P. Yu et al. Positional distribution of transcription factor binding sites in arabidopsis thaliana. *Scientific Reports*, 6(1), apr 2016.
5. J. A. Castro-Mondragon et al. JASPAR 2022: the 9th release of the open-access database of transcription factor binding profiles. *Nucleic Acids Research*, 50(D1):D165–D173, nov 2021.
6. R. C. O'Malley et al. Cistrome and epicistrome features shape the regulatory DNA landscape. *Cell*, 165(5):1280–1292, may 2016.
7. C. E. Grant et al. FIMO: scanning for occurrences of a given motif. *Bioinformatics*, 27(7):1017–1018, feb 2011.
8. J. Friedman et al. Regularization paths for generalized linear models via coordinate descent. *Journal of Statistical Software*, 33(1):1–22, 2010.
9. F. R. Bach. Bolasso: model consistent lasso estimation through the bootstrap. In *Proceedings of the 25th international conference on Machine learning*, pp. 33–40, 2008.
10. N. Meinshausen and P. Bühlmann. Stability selection. *Journal of the Royal Statistical Society: Series B (Statistical Methodology)*, 72(4):417–473, 2010.
11. H. Liu et al. Stability Approach to Regularization Selection (StARS) for High Dimensional Graphical Models. *Advances in neural information processing systems*, 24(2):1432–1440, December 2010.
12. A.-C. Haury et al. TIGRESS: Trustful inference of gene REgulation using stability selection. *BMC Systems Biology*, 6(1), nov 2012.
13. E. R. Miraldi et al. Leveraging chromatin accessibility for transcriptional regulatory network inference in T helper 17 cells. *Genome Res.*, 29(3):449–463, March 2019.
14. F. Petralia et al. Integrative random forest for gene regulatory network inference. *Bioinformatics*, 31(12):i197–i205, jun 2015.
15. V. A. Huynh-Thu et al. Inferring regulatory networks from expression data using tree-based methods. *PloS one*, 5(9):1–10, 2010.
16. E. Scornet. Trees, forests, and impurity-based variable importance. *arXiv preprint arXiv:2001.04295*, 2020.
17. K. K. Nicodemus and J. D. Malley. Predictor correlation impacts machine learning algorithms: implications for genomic studies. *Bioinformatics*, 25(15):1884–1890, may 2009.
18. L. Breiman. Random forests. *Machine learning*, 45:5–32, 2001.
19. A. Greenfield et al. Robust data-driven incorporation of prior knowledge into the inference of dynamic regulatory networks. *Bioinformatics*, 29(8):1060–1067, 2013.
20. C. Skok-Gibbs et al. High-performance single-cell gene regulatory network inference at scale: the inferelator 3.0. *Bioinformatics*, 38(9):2519–2528, feb 2022.
21. F. Bellegarde et al. Signals and players in the transcriptional regulation of root responses by local and systemic n signaling in arabidopsis thaliana. *Journal of Experimental Botany*, 68(10):2553–2565, mar 2017.
22. E. A. Vidal et al. Nitrate in 2020: Thirty years from transport to signaling networks. *The Plant Cell*, 32(7):2094–2119, mar 2020.
23. C.-Y. Cheng et al. Evolutionarily informed machine learning enhances the power of predictive gene-to-phenotype relationships. *Nature Communications*, 12(1), September 2021.
24. J. M. Alvarez et al. Systems approach identifies TGA1 and TGA4 transcription factors as important regulatory components of the nitrate response ofiarabidopsis thaliana/iroots. *The Plant Journal*, 80(1):1–13, aug 2014.
25. T. Kiba et al. Repression of nitrogen starvation responses by members of the arabidopsis garp-type transcription factor nigt1/hrs1 subfamily. *The Plant Cell*, 30(4):925–945, 2018.
26. A. Safi et al. GARP transcription factors repress arabidopsis nitrogen starvation response via ROS-dependent and -independent pathways. *Journal of Experimental Botany*, 72(10):3881–3901, mar 2021.
27. M. D. Brooks et al. Network walking charts transcriptional dynamics of nitrogen signaling by integrating validated and predicted genome-wide interactions. *Nature Communications*, 10(1), apr 2019.
28. C. Marchive et al. Nuclear retention of the transcription factor nlp7 orchestrates the early response to nitrate in plants. *Nature communications*, 4(1):1–9, 2013.
29. J. M. Alvarez et al. Transient genome-wide interactions of the master transcription factor NLP7 initiate a rapid nitrogen-response cascade. *Nature Communications*, 11(1), March 2020.
30. Y. Ueda et al. Nitrate-inducible NIGT1 proteins modulate phosphate uptake and starvation signalling via transcriptional regulation of iSPX/i genes. *The Plant Journal*, 102(3):448–466, January 2020.
31. A. Garg et al. Targeted manipulation of bZIP53 DNA-binding properties influences arabidopsis metabolism and growth. *Journal of Experimental Botany*, 70(20):5659–5671, June 2019.
32. A. Kobayashi et al. INDETERMINATE DOMAIN PROTEIN binding sequences in the 5′-untranslated region and promoter of the SCARECROW gene play crucial and distinct roles in regulating SCARECROW expression in roots and leaves. *Plant Molecular Biology*, 94(1-2):1–13, March 2017.
